# Identification of phytomolecules as isoform and mutation specific PI3K-α inhibitor for protection against breast cancer using e-pharmacophore modeling and molecular dynamics simulations

**DOI:** 10.1186/s13065-024-01317-w

**Published:** 2024-12-18

**Authors:** Ajay Mili, Sumit Birangal, Jyothi Giridhar, Krishnadas Nandakumar, Richard Lobo

**Affiliations:** 1 Department of Pharmacognosy, Manipal College of Pharmaceutical Sciences, Manipal Academy of Higher Education, Manipal, Karnataka 576104 India; 2 Department of Pharmaceutical Chemistry, Manipal College of Pharmaceutical Sciences, Manipal Academy of Higher Education, Manipal, Karnataka 576104 India; 3 Department of Pharmacology, Manipal College of Pharmaceutical Sciences, Manipal Academy of Higher Education, Manipal, Karnataka 576104 India

**Keywords:** Breast cancer, PI3K-α inhibitor, Natural compounds, E-Pharmacophore, Molecular Dynamics

## Abstract

PI3K-α mutation plays a critical role in cancer development, notably in breast cancer, particularly within HR + /HER2- subtypes. These mutations drive tumor growth and survival by activating the PI3K/AKT/mTOR pathway, which is essential for cell proliferation and survival. Our research aimed to identify natural compounds that can inhibit mutant and specific isoforms of PI3K-α to prevent tumor progression. e-Pharmacophore model was generated using Receptor-Ligand complex using the Inavolisib drug (PDB:8EXV) and phase screening was performed using the Molport database of natural compounds. Through molecular docking studies we identified seven promising compounds for further molecular dynamics simulations. Among these, three compounds—STOCK1N-85097, STOCK1N-85998, and STOCK1N-86060—showed significant stability and interaction with PI3K-α. These compounds demonstrated favorable results in several parameters, including RMSD, RMSF, Rg, SASA, PCA, FEL, and total energy evaluations. Therefore, these compounds are projected to function as PI3K-α inhibitors and because of its natural origin it can possess fewer side effects than the conventional medicine, which should be validated by proper in vivo and in vitro models.

**Graphical Abstract:**

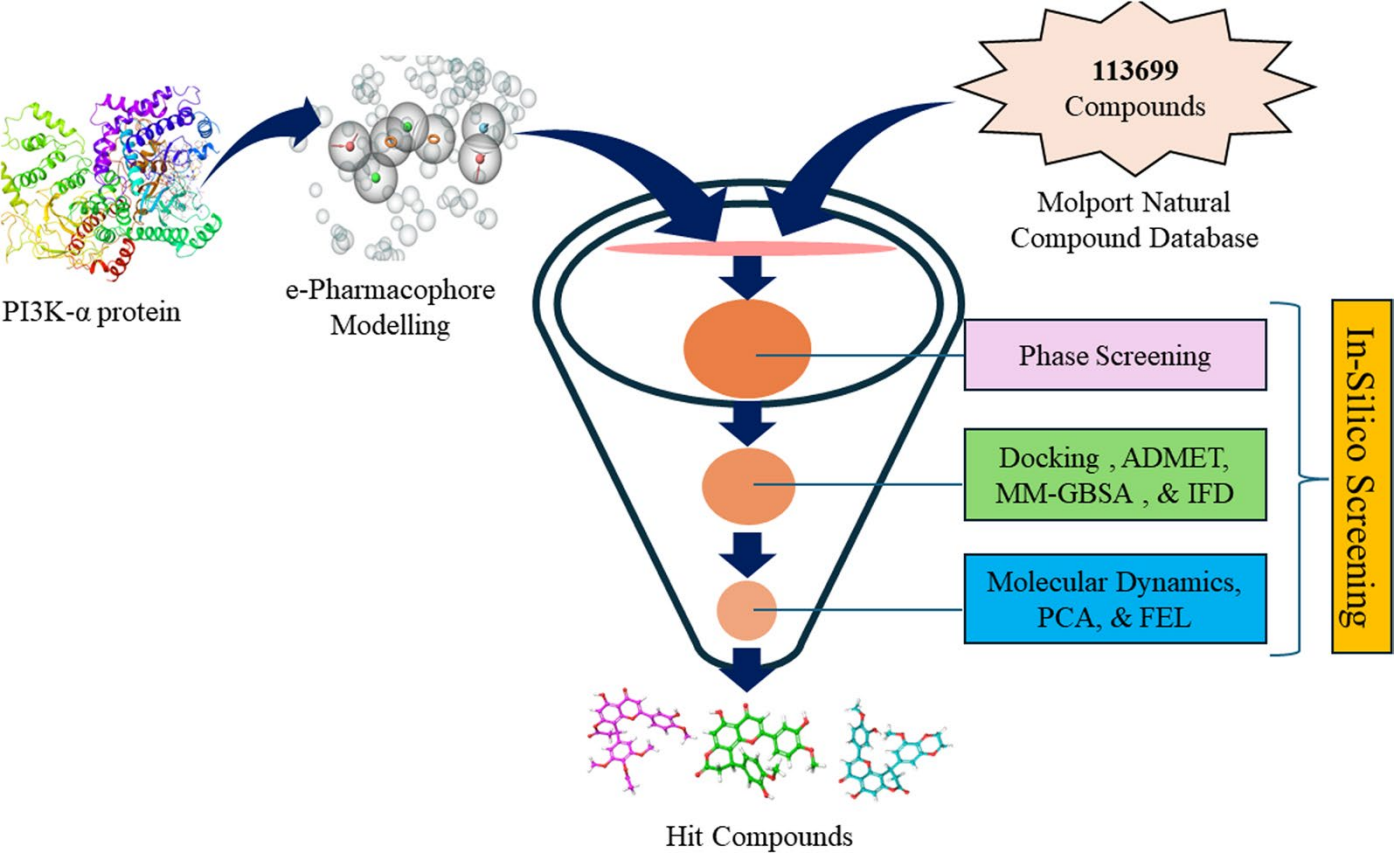

**Supplementary Information:**

The online version contains supplementary material available at 10.1186/s13065-024-01317-w.

## Introduction

Breast cancer is identified by the formation of tumors resulting from uncontrolled growth of abnormal cells within the breast and it is the 2nd most common cancer worldwide. If untreated, these tumors can spread and become fatal. The disease begins in milk ducts or lobules and can be in situ or invasive, causing lumps. Invasive cases can metastasize to lymph nodes or organs, posing a fatal risk. Treatment depends on factors like cancer type, combining surgery, radiation, and medications. In 2022, there were approximately 666,103 death and nearly 2.3 million women were diagnosed with breast cancer with age-standardized rates per 100,000 of 46.8 [1]. This cancer type is the most prevalent, predominantly affecting individuals after puberty and in the later stages of life. About 29% of breast cancers and roughly 40% of hormone receptor positive/human epidermal receptor 2 negative (HR + /HER2-) metastatic breast cancers exhibit abnormal Phosphatidylinositol 3-kinase catalytic 110-KD alpha (PI3K-α) signaling [2].

PI3K-α belongs to Phosphatidylinositol-3 kinase (PI3K) class of lipid kinases. PI3Ks constitute a class of enzymes crucial in cell signaling and controlling diverse cellular functions such as growth, proliferation, survival, and metabolism. PI3Kα is activated through the release of autoinhibition exerted by the nSH2 domain of the p85α regulatory subunit, which triggers conformational changes in the p110α catalytic subunit. PI3K-α works by catalyzing the phosphorylation of Phosphatidylinositol 4,5-bisphosphate (PIP2) to phosphatidylinositol 3,4,5-trisphosphate (PIP3). These generated PIP3 activates the downstream signaling pathway, resulting in cell proliferation, growth, and survival. One of the proteins activated by PIP3 is AKT, which leads toward various cellular processes associated with cancer progression when a disruption or mutation leads to overexpression of PI3K-α which in turn leads to the production of more PIP3 production, resulting in aberrant cellular signaling that promotes the growth of tumors. PI3KCA gene codes for PI3K-α. Around 80% of tumors with mutant PIK3CA possess somatic mutations in the region E545K or H1047R of p110α. Given its crucial role in cancer development, PI3K-α has emerged as a key target for pharmacological efforts in oncology. The goal is to inhibit its function through medication intervention, limiting cancer growth and enhancing the efficacy of treatments. Consequently, there's considerable focus on molecules that act as PI3K-α inhibitors for cancer treatment, with numerous inhibitors progressing to clinical trials [3–7]. For a drug to be an isoform and mutant-specific inhibitor of PI3K-α, it should recognize and bind with amino acid residue GLU859 and preferably recognize and bind with other non-conserved amino acid residues like ARG852, ASN853, SER854, and HIS855 [8–10].

Natural products have exhibited promising potential as compounds for the treatment of cancer, with the capability of displaying activity against prostate cancer [11], lung cancer [12], and breast cancer [13]. The benefits offered by these compounds include fewer side effects and the ability to target multiple pathways involved in cancer progression [14]. The exploration of natural compounds has led to the identification of bioactive molecules capable of specifically targeting cancer cells and disrupting cell signaling pathways [15]. Further investigation and systematic approaches are essential to comprehensively evaluate the therapeutic potential of natural products in cancer treatment.

Computational techniques such as Molecular docking play a crucial role in pharmaceutical research by predicting how potential drug molecules interact with target proteins. This technology enables rapid and cost-effective virtual testing of thousands to millions of compounds. Through precise predictions of how small molecules bind and their affinity for protein binding sites, molecular docking assists in developing and identifying new drugs [16]. Several studies have employed computational techniques to identify potential compounds that act as selective cyclin-dependent kinase 2 inhibitor for treating cancer [17], DprE1 inhibitors for tuberculosis treatment [18], and γ-aminobutyric acid receptor antagonist [19]. Hence, in this research, computational techniques like e-pharmacophore modeling, ligand docking, and molecular dynamic simulation studies are being utilized to identify natural products as isoform and mutation-specific inhibitors of PI3K-α.

### Methods

The graphical user interface (GUI) of Schrödinger 2024–2, Maestro version 14 was used for all computational calculations and analyses such as the Phase module "Develop hypothesis model" and "Hypothesis validation" was used for developing Pharmacophore hypothesis and Validation, the Glide module "Ligand docking" was used for Molecular docking, the Prime module "MM-GBSA" was used for MM-GBSA calculation, the task pane Receptor-based virtual screening "Induced fit docking" for Induced fit docking studies. "QikProp" for ADME analysis and "Desmond" for Molecular dynamic simulation studies. Additionally, Geo-Measures: A PyMOL plugin was used for principal component analysis (PCA), Free energy landscape (FEL) and Radius of gyration [20].

### Protein preparation, grid generation and Ligand preparation

The Protein Data Bank (PDB) served as the source for downloading the PI3K-α protein structure, which houses numerous experimentally determined protein structures. However, indiscriminate utilization of all available models can yield unreliable results and interpretations due to varying model quality. Hence, stringent criteria were employed in selecting an appropriate protein crystal structure like a high-resolution structure was required to ensure accurate representation, completeness of the active site and side chains to prevent inaccurate binding interaction analysis, the presence of a co-crystallized ligand for precise delineation of the active site's location. Additionally, the chosen PDB’s root mean square deviation (RMSD) had to be minimal to confirm the structural stability and accuracy of the protein. The selected protein is downloaded and prepared using the Schrödinger suite module "Protein Preparation Workflow". The process involves importing, preprocessing, optimize H-bond assignment, minimize, and delete water. OPLS4 (Optimized potential for liquid stimulation) force field was used for minimization of energy and to generate protein low energy state. water molecules beyond 5 Å were deleted, and the heteroatoms stage was generated. The "Receptor grid generation" panel used to build a grid surrounding the ligand at coordinates X (– 17.25), Y (13.63), and Z (29.42) while preserving all functional residues inside the grid in the default configuration [21–23].

The Molport database for Natural products was selected for the docking studies. The database consists of 113,699 molecules. The LigPrep tool of the Schrödinger suite was used to prepare molecules for docking. The molecules was prepared in OPLS4 forcefield with ionization kept at pH 7.4 ± 0.0 using Epik and only 1 stereoisomers were generated while keeping rest of the setting in default [21–23].

### e-pharmacophore based screening

#### Generation of e-pharmacophore model

For the development of e-pharmacophore model, the phase module "Develop hypothesis model" was used for generating the hypothesis where pharmacophore model was created using the “Receptor-ligand complex” with Auto (E-Pharmacophore) Method keeping 7 as Maximum number of features, 2 Å as Minimum feature-feature distance and 4 Å keeping Minimum feature-feature distance for feature of the same type I the feature section of the hypothesis settings. In the Excluded Volumes section, Create receptor-based excluded volume shell was ticked with Radii size selected as Van der Waals radii of receptor atoms, Radii scaling factors was selected at Fixed scaling factor of 0.50, the surfaces of receptor atoms were ignored which are within 2 Å of the ligand surface and 5 Å is kept as limit for excluded volume shell thickness.

#### e-pharmacophore model validation

The generated pharmacophore model quality was assessed using Test set validation and Güner-Henry (GH) scoring method [24]. Drug-Like Ligand Decoys Set from Schrödinger website (https://www.schrodinger.com/products/glide#block-2294) was downloaded. 15 molecules with reported activity on PI3K-α with Ki value in the range of 0.026–0.681 nM were identified from literature search and used as active set (Supplementary file Table S1). These sets of active and decoy were used for hypothesis validation. The Schrödinger suite's Phase module "Hypothesis validation" was used for validating the hypothesis.

#### Phase screening

The e-Pharmacophore model selected after validation was used for screening Molport natural product database. A well validated model helps in the identification of molecules with similar pharmacophoric properties organized in similar relative orientations. Molecules that best fit the query and are returned as hits. The Phase module's "Ligand and Database screening" was used for phase screening using the Molport database for Natural product with Hypothesis matching 6 out of 7 features of the generated e-Pharmacophore model.

### Molecular docking and free ligand binding energy (MM-GBSA)

The Schrödinger suite GLIDE module “Ligand Docking” was used for molecular docking studies. Ligands which passed through Phase Screening process were used in the study. Firstly, the prepared receptor grid was selected, the prepared ligand was selected in the Ligand tab while keeping the scaling factor as 0.80 with partial charge cutoff at 0.15 for van der Waals radii and also ligands having more than 500 atoms, and 100 rotatable bonds was not docked or scored. In the Settings section, Precision was selected initially as High throughput virtual screening (HTVS) followed by Standard precision (SP) mode, top 10 percent of the hit was again docked using Extra precision (XP) mode, this process helps in avoid false positive results with Ligand sampling kept as flexible. The Constraints section is kept on default. In the Output section, settings was kept in default with post-docking minimization and compute RMSD to input ligand geometries selected [21–23].

Generalized Born and surface area solvation (MM-GBSA) is used for calculating the absolute binding affinities of ligand–protein complex by estimating binding-free energy. The Prime module's MM-GBSA panel was used for calculating the MM-GBSA keeping the settings in default [25].

The Formula for MM-GBSA calculation in given below:

$$\Delta G_{(binding)} = \, \Delta E_{(MM)} + \, \Delta G_{(SA)} + \, \Delta G_{(solvation)}$$

where, ΔG_(SA)_ is difference of the PI3K-α-Inhibitor complex’s surface energies and the sum of unbounded PI3K-α protein and inhibitor surface area energies. ΔE_(MM)_ is difference of the PI3K-α-Inhibitor complex’s minimized energies and the sum of unbounded PI3K-α protein and inhibitor energies. ΔG_(solvation)_ difference of the PI3K-α-Inhibitor complex’s solvation energies and the sum of unbounded PI3K-α protein and inhibitor solvation energies.

### ADMET predictions

The top hits based on MM-GBSA, docking score and interaction, were selected for further analysis. The QikProp tool of the Schrödinger suite was used for predicting ADME parameters such as QPlogS (predicted aqueous solubility), QPlogBB (Predicted brain/blood partition coefficient), QPlogKhsa (prediction of binding to human serum albumin), QPlogPo/w (predicted octanol/water coefficient). Bioavailability and drug-likeness using Lipinski's rule of five by using SwissADME (http://www.swissadme.ch/) [26]. "admetSAR" was used for toxicity profiling [27].

### Induced fit docking (IFD)

On the basis of XP docking score & interaction formed, MM-GBSA and ADMET prediction, leads will be selected for IFD. IFD considers the flexibility of both the receptor and the ligand during docking. It explains the dynamic changes in the receptor's structure that occur when a ligand binds to it. It enhances the accuracy of predicting binding interactions by allowing the receptor to change its binding location to better match the ligand. By considering the dynamic nature of protein–ligand interactions, this technique improves the success rate of structure-based drug design.

The Extra precision-Induced Fit docking module of the Schrödinger suite was used for this assessment. A total of 20 different poses were generated and the rest of the setting was kept in default [28–30].

### Molecular dynamic simulations (MDS)

Molecular dynamic simulation (MDS) studies were employed for examining the functioning and the dynamics of ligand–protein complexes. Molecular docking and IFD studies don't mimic the body biological environment as the protein and ligands were suspended in water, therefore MDS is used for addressing this concern. Molecules showing good bond retention and IFD docking score were selected for undergoing MDS for a duration of 100 ns. The MDS process involves Desmond’s System builder module where the protein–ligand complex was immersed into a simple point charge (SPC) solvent model and Na^+^ used as counterion. The boundary condition was kept as orthorhombic form and the buffer box size was calculated using Buffer method with dimensions set at a, b, & c at 10 Å and angles α, β & γ at 90°. OPLS4 force field was used, and Minimization tool was used for system minimization. Molecular Dynamics module of Desmond was selected for MDS, where simulation time was set at 100 ns, with trajectory recording interval at 100 picoseconds with 1000 frames. Ensemble class was set at NPT (constant particle number, pressure 1.01325 bar, and temperature 300 K). In the integration tab, RESPA integrator setting was kept at Time step (fs) bonded at 2 with near and far steps at 2 and 6. In the Ensemble tab, Thermostat method was set to Nose–Hoover chain with relaxation time of 1 picosecond and 1 as number of groups. The Martyna-Tobias-Klein, Barostat method was used with relaxation time of 2 picoseconds and isotropic as coupling style. The Coulombic cutoff radius was set at 9 Å with no position restraints were set, and Seed is kept at Random [31–33]. The force field used was OPLS4. Finally, the report was generated using Simulation Interaction Diagram tool. Post MDS studies including Root mean square deviation (RMSD), Root mean square fluctuation (RMSF), Solvent accessible surface area (SASA), Radius of gyration (Rg) Principal component analysis (PCA), Free energy landscape (FEL), Hydrogen bond analysis, Thermal Binding free energy (MM‑GBSA dG bind) and Total energy were also assessed.

## Result and discussion

### Hypothesis generation, validation and phase screening

**Fig. 1:**
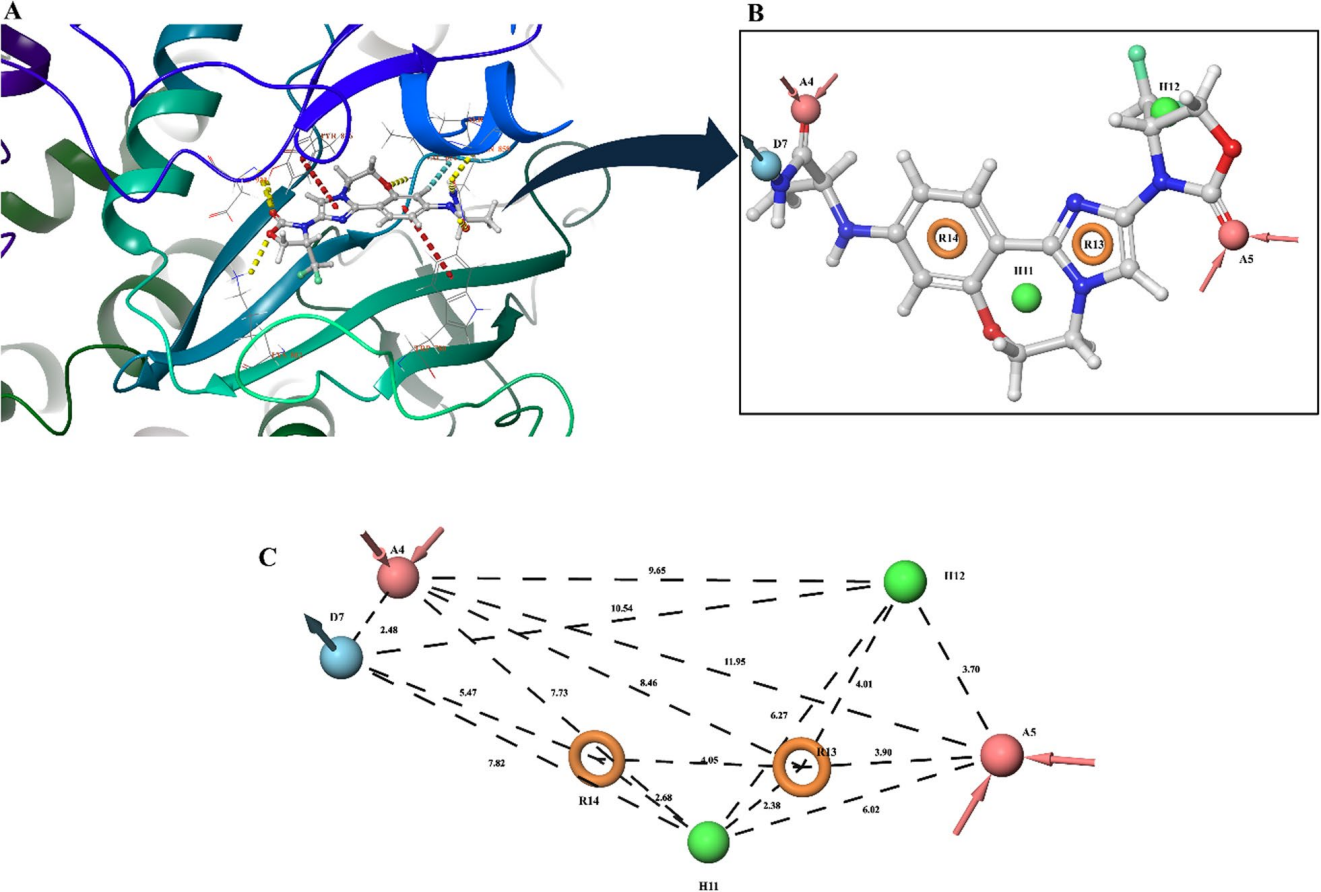
**A** Protein structure with Ligand bound at active site. **B** e-Pharmacophore model **C** Inter-site distances (Å) between the pharmacophoric points of model. Pink Color is Hydrogen bond acceptor (A); Green color represents Hydrophobic atom (H); Blue Color represents Hydrogen bond donor (D); Yellow color represents Aromatic ring (R)

For generate the hypothesis, we identified 72 PDB entries of PI3K-α in complex with a co-crystallized ligand. Based on the selection criteria outlined in the methods section, PDB ID 8EXV was chosen. This entry was selected due to its low RMSD of 0.1557 Å, a high X-ray crystallographic resolution of 2.48 Å, and the presence of the co-crystallized ligand Inavolisib in the active site. Additionally, the co-crystallized ligand Inavolisib (X3N) showed an IC50 value of 0.038 nmol and is also under clinical trial Phase-3 for clinical indication of HR + , HER2-negative, PIK3CA mutated mBC post CDK4/6i therapy [34]. Its mechanism of action involves binding to the ATP binding site of PI3K by making Hydrogen bonding with TYR836, VAL851, GLN859 and ASP933 amino acid residues and π-π stacking with TRP780 and TYR836 amino acid residues, thereby effectively inhibiting the phosphorylation process that converts PIP2 to PIP3. Importantly, Inavolisib demonstrates greater selectivity for mutant PI3Kα than the wild-type form [35]. PDB-8EXV complexed with Inavolisib was used for generating the Ligand-Receptor complex e-pharmacophore model. The presence of pharmacophoric features such as hydrogen acceptors, hydrophobic regions, hydrogen donors, and aromatic rings is beneficial because these elements contribute to stronger and more specific binding interactions between the ligand and the target protein. Hydrogen acceptors and donors help form stable hydrogen bonds, hydrophobic regions enhance the fit within nonpolar pockets, and aromatic rings can participate in π-π stacking and hydrophobic interactions, all of which improve the ligand's affinity and specificity for its target. The model generated contains 7 pharmacophoric features including two hydrogen acceptor regions (A4 & A5), two hydrophobic regions (H11 & H12), one donor (D7) and two aromatic rings (R13 & R14) (Fig. 1).

Now, the e-Pharmacophore model was then validated by assessing its capability to recognize active molecules from the dataset containing a set of actives and decoys. The pharmacophore model's validation was carried out using Güner–Henry (GH) and Enrichment Factor (EF) metrics to assess its capability in distinguishing between active and inactive compounds. The GH method, or Goodness of Hit list, combines two metrics: the percentage of active compounds identified and the proportion of actives in the hit list. The EF measures how much the hit list is enriched compared to the overall database. For this validation, a decoy dataset consisting of 1000 inactive compounds and 15 active compounds was created. The generated model was able to identify 100% of the actives and assumed 6 decoys as actives from 1000 decoys. It shows a Goodness of hit (GH) score of 0.78, whereas effective pharmacophore model is expected to have a GH score greater than 0.60 [36]. The Enrichment factor (EF) score of 48.33 gives an idea about the number of times the model was able to identify actives in the set of decoys, i.e., it can screen actives 48.33 times more than the decoys. Additional validation metrics, including the percentage of active compounds, false positives, and false negatives, are detailed in Table 1. The result indicates that the generated model can be used for phase screening.

**Table 1:** Validation parameters of the e-Pharmacophore model

Parameter	e-Pharmacophore model
Total compounds in the database (D)	1015
Number of actives in the database (A)	15
Number of hits (H_t_)	21
Number of actives in the hit list (H_A_)	15
Percentage Yield of actives (Ya %) = (HA/ H_t_) × 100	71.43%
Percentage Ratio of actives = (H_A_/A) × 100	100%
Enrichment factor (EF) = (H_A_ x D)/( H_t_ x A)	48.33
True Positive (TP)	15
True Negative (TN) = D—H_t_	994
False Negatives (FN) = A—H_A_	0
False Positives (FP) = H_t_ – H_A_	6
Sensitivity (Se) = TP/(TP + FN)	1
Specificity (Sp) = TN/(TN + FP)	0.99
Goodness of hit (GH) score = [(3/4 × Ya) + (1/4 × Se)] x Sp	0.78

Phase screening using the validated e-Pharmacophore model was done using the Molport database for Natural products which contain 113,669 molecules. Ligands were prepared as mentioned in the Ligand preparation section. In the Ligand and Database screening module, the validated hypothesis was selected in the hypothesis section, Matches was selected at 6 of 7 while keeping the screening settings at default. A total of 5600 hits was found in the initial screening which has 6 of 7 pharmacophoric features similar to Inavolisib. The hit was compiled and used for Molecular docking studies.

### Molecular docking studies, MM-GBSA, ADMET and induced fit docking (IFD)

The 5600 hits obtained after Phase screening was docked with the protein structure of PI3K-α (PDB-8EXV). Firstly, HTVS docking mode was used for identifying interactions and docking scores. Which was followed by SP-docking mode, where the top 10 percent of the hits after HTVS docking mode was selected. Based on the SP-docking score and interaction, the top 10 percent of the ligands were again selected for XP docking mode. Based on XP docking mode score, interaction with protein and MM-GBSA, 25 molecules namely STOCK1N-113116, STOCK1N-81073, STOCK1N-51145, STOCK1N-59405, STOCK1N-84648, STOCK1N-46158, STOCK1N-85097, STOCK1N-84443, STOCK1N-64228, STOCK1N-84098, STOCK1N-85873, STOCK1N-92951, STOCK1N-92949, STOCK1N-40367, STOCK1N-85433, STOCK1N-1935, STOCK1N-81980, STOCK1N-85851, STOCK1N-84154, STOCK1N-85998, STOCK1N-86351, STOCK1N-88743, STOCK1N-42002, STOCK1N-86060 and STOCK1N-83010 having docking score in the range of − 12.144 to − 16.683 kcal/mol was selected for futher screening (Table 2).

**Table 2:** Docking score, MMGBSA and interaction with protein of the 25 selected hits and the Co-crystalized Ligand

Compound ID	Structure	MM-GBSA (kcal/mol)	Docking score (kcal/mol)	Interaction with protein
Inavolisib		− 102.74	− 13.007	**H-bond:** TYR836, VAL851, GLN859 & ASP933 **π-π stacking:** TRP780 & TYR836
STOCK1N-113116		− 72.85	− 16.683	**H-bond:** ARG770, SER774, ASP810, TYR836, VAL851, SER854, GLN859, SER919 & ASP933
STOCK1N-81073		− 53.4	− 14.446	**H-bond:** SER774, LYS802, ASP810, TYR836 & ASP933
STOCK1N-51145		− 57.47	− 13.683	**H-bond:** VAL851, GLN859, SER919 & ASP933
STOCK1N-59405		− 60.74	− 13.614	**H-bond:** ARG770, TRP780, GLU798, VAL851, ASN853, SER854, GLN859 & ASP933 **Salt bridge:** ARG770 **π-π stacking:** TRP780 & TYR836
STOCK1N-84648		− 65.78	− 13.536	**H-bond:** ALA775, LYS802, VAL851, SER854 & ASP933 **π-π stacking:** TRP780 & TYP836
STOCK1N-46158		− 37.92	− 13.513	**H-bond:** VAL851 & ASP933 **Salt bridge:** ASP805 & ASP933 **π-π stacking:** TYR836
STOCK1N-85097		− 53.02	− 13.458	**H-bond:** SER774, LYS802, VAL851 & SER854 **π-π stacking:** TRP780 & TYR836
STOCK1N-84443		− 48.34	− 13.32	**H-bond:** SER774, LYS802, GLU849, VAL851, SER854 & GLN859 **π-π stacking:** TRP780 & TYR836
STOCK1N-64228		− 55.47	− 13.183	**H-bond:** ARG770, VAL851, ASN853, GLN859 & LYS802 **π-π stacking:** TRP780
STOCK1N-84098		− 59.56	− 12.857	**H-bond:** SER773, SER774 & VAL851 **Salt bridge:** ARG770 **π-cation stacking:** ARG770
STOCK1N-85873		− 55.74	− 12.773	**H-bond:** ALA775, LYS802, VAL851, SER854 & GLN859 **π-π stacking:** TRP780 & TYR836
STOCK1N-92951		− 54.55	− 12.667	**H-bond:** ALA775, LYS802, VAL851 & SER919
STOCK1N-92949		− 65.7	− 12.641	**H-bond:** LYS802, VAL851, ASN853, HIS855, GLN859 & ASP933
STOCK1N-40367		− 58.95	− 12.596	**H-bond:** ARG770, VAL851, ASN853, SER854 & GLN859 **Salt bridge:** ARG770
STOCK1N-85433		− 58.48	− 12.371	**H-bond:** LYS802, TYR836, VAL851 & SER854 **π-π stacking:** TRP780
STOCK1N-1935		− 48.91	− 12.348	**H-bond:** GLU849 & VAL851 **π-π stacking:** TRP780 & TYR836
STOCK1N-81980		− 46.92	–12.337	**H-bond:** GLU849, VAL851 & ASP933 **π-π stacking:** TYR836
STOCK1N-85851		− 41.61	− 12.292	**H-bond:** SER774 **π-π stacking:** TYR836 **Salt bridge:** ARG770
STOCK1N-84154		− 56.81	− 12.289	**H-bond:** LYS802, VAL851 & SER854 **π-π stacking:** TRP780 & TYR836
STOCK1N-85998		− 59.22	− 12.273	**H-bond:** LYS802, VAL851, SER854 & GLN859 **π-π stacking:** TRP780 & TYR836
STOCK1N-86351		− 53.71	− 12.241	**H-bond:** VAL851, SER854 & LYS802 **π-π stacking:** TRP780 & TYR836
STOCK1N-88743		− 72.47	− 12.183	**H-bond:** VAL851, GLN859 & SER919 **π-π stacking:** TRP780 & TYR836
STOCK1N-42002		− 50.58	− 12.166	**H-bond:** ALA775, VAL851 & ASP933 **π-π stacking:** TYR836
STOCK1N-86060		− 58.79	− 12.152	**H-bond:** LYS802, VAL851, SER854 & GLN859 **π-π stacking:** TRP780 & TYR836
STOCK1N-83010		− 67.11	− 12.144	**H-bond:** ALA775, GLU849, ASN853, SER854, GLN859 & ASP933

The Inavolisib form Hydrogen bond with TYR836, VAL851, GLN859 & ASP933 and π-π stacking with TRP780 & TYR836 amino acid residue of PI3K-α protein. The hit STOCK1N-113116 form Hydrogen bonds with ARG770, SER774, ASP810, TYR836, VAL851, SER854, GLN859, SER919 & ASP933 amino acid residue of PI3K-α protein. The hit STOCK1N-81073 form Hydrogen bond with SER774, LYS802, ASP810, TYR836 & ASP933 amino acid residue of PI3K-α protein. The hit STOCK1N-51145 form Hydrogen bond with VAL851, GLN859, SER919 & ASP933 amino acid residue of PI3K-α protein. The hit STOCK1N-59405 forms Hydrogen bond with ARG770, TRP780, GLU798, VAL851, ASN853, SER854, GLN859 & ASP933, Salt bridge with ARG770 and π-π stacking with TRP780 & TYR836 amino acid residue of PI3K-α protein. The hit STOCK1N-84648 form Hydrogen bond with ALA775, LYS802, VAL851, SER854 & ASP933 and π-π stacking TRP780 & TYP836 amino acid residue of PI3K-α protein. The hit STOCK1N-46158 form Hydrogen bond with VAL851 & ASP933, Salt bridge with ASP805 & ASP933 and π-π stacking with TYR836 amino acid residue of PI3K-α protein. The hit STOCK1N-85097 form hydrogen bond with SER774, LYS802, VAL851 & SER854 and π-π stacking with TRP780 & TYR836 amino acid residue of PI3K-α protein. The hit STOCK1N-84443 form hydrogen bond with SER774, LYS802, GLU849, VAL851, SER854 & GLN859 and π-π stacking with TRP780 & TYR836 amino acid residue of PI3K-α protein. The hit STOCK1N-64228 forms Hydrogen bond with ARG770, VAL851, ASN853, GLN859 & LYS802 and π-π stacking with TRP780 amino acid residue of PI3K-α protein. The hit STOCK1N-84098 forms Hydrogen bond with SER773, SER774 & VAL851, Salt bridge with ARG770 and π-cation stacking with ARG770 amino acid residue of PI3K-α protein. The hit STOCK1N-85873 forms Hydrogen bond with ALA775, LYS802, VAL851, SER854 & GLN859 and π-π stacking with TRP780 & TYR836 amino acid residue of PI3K-α protein. The hit STOCK1N-92951 form Hydrogen bond with ALA775, LYS802, VAL851 & SER919 amino acid residue of PI3K-α protein. The hit STOCK1N-92949 form Hydrogen bond with LYS802, VAL851, ASN853, HIS855, GLN859 & ASP933 amino acid residue of PI3K-α protein. The hit STOCK1N-40367 form Hydrogen bond with ARG770, VAL851, ASN853, SER854 & GLN859, and Salt bridge with ARG770 amino acid residue of PI3K-α protein. The hit STOCK1N-85433 form Hydrogen bond with LYS802, TYR836, VAL851 & SER854 and π-π stacking with TRP780 amino acid residue of PI3K-α protein. The hit STOCK1N-1935 form Hydrogen bond with GLU849 & VAL851, and π-π stacking with TRP780 & TYR836 amino acid residue of PI3K-α protein. The hit STOCK1N-81980 form Hydrogen bond with GLU849, VAL851 & ASP933, and π-π stacking with TYR836 amino acid residue of PI3K-α protein. The hit STOCK1N-85851 form Hydrogen bond with SER774, π-π stacking with TYR836, and Salt bridge with ARG770 amino acid residue of PI3K-α protein. The hit STOCK1N-84154 form H-bond with LYS802, VAL851 & SER854, and π-π stacking with TRP780 & TYR836 amino acid residue of PI3K-α protein. The hit STOCK1N-85998 form Hydrogen bond with LYS802, VAL851, SER854 & GLN859, and π-π stacking with TRP780 & TYR836 amino acid residue of PI3K-α protein. The hit STOCK1N-86351 for Hydrogen bond with VAL851, SER854 & LYS802, and π-π stacking with TRP780 & TYR836 amino acid residue of PI3K-α protein. The hit STOCK1N-88743 form Hydrogen bond with VAL851, GLN859 & SER919, and π-π stacking with TRP780 & TYR836 amino acid residue of PI3K-α protein. The hit STOCK1N-42002 form Hydrogen bond with ALA775, VAL851 & ASP933, and π-π stacking with TYR836 amino acid residue of PI3K-α protein. The hit STOCK1N-86060 form Hydrogen bond with LYS802, VAL851, SER854 & GLN859, and π-π stacking with TRP780 & TYR836 amino acid residue of PI3K-α protein. The hit STOCK1N-83010 form Hydrogen bond with ALA775, GLU849, ASN853, SER854, GLN859 & ASP933 amino acid residue of PI3K-α protein. It has been observed that most of the compound shows interactions with the amino acid residues (TYR836, VAL851, GLN859 & ASP933, TRP780 and TYR836) of PI3K-α similar to the Inavolisib (co-crystalized ligand). The 2D interaction diagram is shown in Supplementary file Table S2.

The MM-GBSA value is in the range of the 25 molecules are in the range of − 37.92 to − 72.85 kcal/mol (Table 2), greater the negative value greater is the stability of the docked compound in the protein–ligand complex.

During the process of drug design, the criteria of drug-like properties and ADMET properties are of utmost importance. Drug-like properties was measured using Lipinski rule filter (Molecular weight (MW) < 500 Da, Number of hydrogen bond acceptors < 10, Number of hydrogen bond donors < 5, Calculated n-octanol–water partition coefficient (Clog P) < 5) where we observed that more than 5 compounds (STOCK1N-113116, STOCK1N-81073, STOCK1N-59405, STOCK1N-64228, STOCK1N-83010) has shown a violation of more than 3 factors, four compounds (STOCK1N-84648, STOCK1N-92951, STOCK1N-92949, STOCK1N-86060) has shown a violation of 2 factors and sixteen compounds (STOCK1N-51145, STOCK1N-46158, STOCK1N-85097, STOCK1N-84443, STOCK1N-84098, STOCK1N-85873, STOCK1N-40367, STOCK1N-85433, STOCK1N-1935, STOCK1N-81980, STOCK1N-85851, STOCK1N-84154, STOCK1N-85998, STOCK1N-86351, STOCK1N-88743, STOCK1N-42002) have shown a violation of 0 or 1 (Table 3).

**Table 3:** Drug-likeness and ADME profile of the selected compounds

Compound ID	mol wt	donorHB	accptHB	QPlogS	QPlogPo/w	QPlogBB	QPlogKhsa	%OralAbsorption	Lipinski RuleOf5	Bioavailability Score
STOCK1N-113116	770.69	10	27.35	− 2.08	− 3.2	− 5.3	− 1.8	0	3	0.17
STOCK1N-81073	624.55	8	20.55	− 2.47	− 1.5	− 3.7	− 1.2	0	3	0.17
STOCK1N-51145	477.51	5	14.5	− 2.27	− 0.3	− 2.8	− 1.2	51	0	0.55
STOCK1N-59405	534.47	6	15.75	− 3.50	− 0.3	− 4.5	− 1.1	0	3	0.17
STOCK1N-84648	533.49	3	10.25	− 5.07	1.9	− 3.3	0.1	25	2	0.17
STOCK1N-46158	384.47	1	7.5	− 3.82	2.6	− 0.5	0.02	84	0	0.55
STOCK1N-85097	476.43	2	7.75	− 4.64	2.5	− 1.9	0.4	71	0	0.55
STOCK1N-84443	446.41	2	7	− 5.65	2.6	− 2.3	0.4	71	0	0.55
STOCK1N-64228	696.61	7	21.6	− 3.89	− 0.9	− 5.1	− 1.1	0	3	0.17
STOCK1N-84098	568.53	3	8.5	− 5.94	3.4	− 2.7	0.6	56	1	0.55
STOCK1N-85873	592.60	1	7.75	− 6.26	4.9	− 1.6	1.1	82	1	0.55
STOCK1N-92951	492.43	5	13.75	− 2.87	0.1	− 2.6	− 0.7	27	2	0.17
STOCK1N-92949	494.40	7	14.5	− 2.58	− 1.3	− 3.8	− 0.9	1	2	0.17
STOCK1N-40367	499.56	4.25	8.75	− 3.86	2.3	− 2.6	− 0.4	51	0	0.11
STOCK1N-85433	488.45	1	7.75	− 5.88	3.5	− 1.5	0.6	87	0	0.55
STOCK1N-1935	366.41	3	4	− 4.53	3.4	− 0.8	0.5	96	0	0.55
STOCK1N-81980	396.39	2	6.25	− 4.81	2.4	− 2.1	0.3	69	0	0.55
STOCK1N-85851	421.36	4	7.5	− 4.17	1.8	− 2.7	− 0.2	49	0	0.11
STOCK1N-84154	484.46	1	7	− 6.28	3.8	− 1.6	0.7	87	0	0.55
STOCK1N-85998	518.47	1	8.5	− 5.47	3.5	− 1.4	0.5	74	1	0.55
STOCK1N-86351	520.49	1	8.5	− 5.70	3.7	− 1.7	0.6	74	1	0.55
STOCK1N-88743	460.43	2	8	− 5.57	3.2	− 1.7	0.4	85	0	0.55
STOCK1N-42002	456.49	1.5	8.5	− 4.8	2.4	− 2.5	− 0.4	56	0	0.56
STOCK1N-86060	548.50	1	10.25	− 5.52	3.1	− 1.9	0.4	53	2	0.17
STOCK1N-83010	564.49	7	18.1	− 3.04	− 1.4	− 4.2	− 1.1	0	3	0.17

**QPlogKhsa** prediction of binding to human serum albumin, **QPlogBB** predicted brain/blood partition coefficient, **QPlogS** predicted aqueous solubility, **QPlog Po/w** predicted octanol/water partition coefficient

One of the crucial factors is the oral absorption percentage of the compounds, it was observed that sixteen compounds (STOCK1N-51145, STOCK1N-46158, STOCK1N-85097, STOCK1N-84443, STOCK1N-85873, STOCK1N-85433, STOCK1N-1935, STOCK1N-81980, STOCK1N-84154, STOCK1N-85998, STOCK1N-86351, STOCK1N-88743, STOCK1N-42002, & STOCK1N-86060) shows >50 percent oral obsorption with three compounds, STOCK1N-84648, STOCK1N-92951, & STOCK1N-85851 shows absorption of 25, 27 and 49% oral absorption, respectively while six compounds (STOCK1N-113116, STOCK1N-81073, STOCK1N-59405, STOCK1N-64228, STOCK1N-92949, & STOCK1N-83010) shows 0 or 1 percent oral absorption. Fourteen compounds show Bioavailability of more than 50% while eleven compounds namely STOCK1N-113116, STOCK1N-81073, STOCK1N-59405, STOCK1N-84648, STOCK1N-64228, STOCK1N-92951, STOCK1N-92949, STOCK1N-40367, STOCK1N-85851, STOCK1N-86060, & STOCK1N-83010 show less than 20% bioavailability. Additional properties, namely H-bond donor or acceptor and QPlogPo/w, should fall within the specific range of 0.0 to 6.0, 2.0 to 20.0 and − 2.0 to 6.5, respectively. Furthermore, the predicted pharmacokinetic parameters such as QPlogS of all the compounds were in the range of − 6.28 to − 2.08 which is well within the recommended range of − 6.5 to 0.05. The QPlogKhsa values of the 24 compounds ranged from – 1.2 to 1.1 and with compound STOCK1N-113116 get a score of – 1.8 which falls beyond the recommended range of − 1.5 to 1.5. The normal range of QPlogBB is from − 3.0 to 1.2, and all the compounds were found to be within the range of − 2.2 to − 0.2. The predicted toxicity profiles indicate that all compounds exhibit a medium risk of acute oral toxicity. With the exception of STOCK1N-1935, all other compounds show minimal risk of crossing the blood–brain barrier. Four compounds—STOCK1N-46158, STOCK1N-1935, STOCK1N-85851, and STOCK1N-42002—are associated with a high risk of hepatotoxicity, whereas the remaining compounds demonstrate a lower risk in this regard. Twelve compounds, including STOCK1N-113116, STOCK1N-81073, STOCK1N-51145, STOCK1N-59405, STOCK1N-92951, STOCK1N-92949, STOCK1N-40367, STOCK1N-85433, STOCK1N-1935, STOCK1N-81980, STOCK1N-85851, and STOCK1N-42002, exhibit a lower risk of inhibiting the Human Ether-a-go-Go-Related Gene (hERG). The remaining compounds show a higher risk of hERG inhibition. Five compounds—STOCK1N-113116, STOCK1N-81073, STOCK1N-59405, STOCK1N-92951, and STOCK1N-92949—present a lower risk of mitochondrial toxicity, while the others demonstrate a higher risk. All compounds show a lower risk of nephrotoxicity. However, only two compounds, STOCK1N-46158 and STOCK1N-83010, exhibit a lower risk of respiratory toxicity, while the rest are associated with a higher risk (Table 4).

**Table 4:** Toxicity profile of the selected compounds

Compound ID	Acute Oral Toxicity	Blood Brain Barrier	Hepatotoxicity	Human Ether-a-go-go-Related Gene inhibition	Mitochondrial toxicity	Nephro-toxicity	Respiratory toxicity
STOCK1N-113116	III	–	–	–	–	–	+
STOCK1N-81073	III	–	–	–	–	–	+
STOCK1N-51145	III	–	–	–	+	–	+
STOCK1N-59405	III	–	–	–	–	–	+
STOCK1N-84648	III	–	–	+	+	–	+
STOCK1N-46158	III	–	+	+	+	–	–
STOCK1N-85097	III	–	–	+	+	–	+
STOCK1N-84443	III	–	–	+	+	–	+
STOCK1N-64228	III	–	–	+	+	–	+
STOCK1N-84098	III	–	–	+	+	–	+
STOCK1N-85873	III	–	–	+	+	–	+
STOCK1N-92951	III	–	–	–	–	–	+
STOCK1N-92949	III	–	–	–	–	–	+
STOCK1N-40367	III	–	–	–	+	–	+
STOCK1N-85433	III	–	–	–	+	–	+
STOCK1N-1935	III	+	+	–	+	–	+
STOCK1N-81980	III	–	–	–	+	–	+
STOCK1N-85851	III	–	+	–	+	–	+
STOCK1N-84154	III	–	–	+	+	–	+
STOCK1N-85998	III	–	–	+	+	–	+
STOCK1N-86351	III	–	–	+	+	–	+
STOCK1N-88743	III	–	–	+	+	–	+
STOCK1N-42002	III	–	+	–	+	–	+
STOCK1N-86060	III	–	–	+	+	–	+
STOCK1N-83010	III	–	–	+	+	–	–

− low risk, + high risk and III medium risk

Induced fit docking (IFD) score of the 25 selected compounds ranges from − 1987.21 to − 1915.39 kcal/mol. Based on the interaction retained during the IFD and ADMET profile, 7 ligands, namely, STOCK1N-85097, STOCK1N-84443, STOCK1N-85433, STOCK1N-84154, STOCK1N-85998, STOCK1N-88743, & STOCK1N-86060 were selected for molecular dynamics simulation studies. IFD score and 3D interaction image is provided in Supplementary file Table S3.

### Molecular dynamic simulation (MDS) studies

In molecular dynamics simulations (MDS), convergence is assessed through the analysis of several key metrics. The root mean square deviation (RMSD) is utilized to evaluate the stability of structural deviations from the initial configuration. The root mean square fluctuation (RMSF) measures the consistency of atomic fluctuations across the simulation. The total energy of the system is monitored to ensure it stabilizes, indicating that the system’s energy is equilibrated. Principal component analysis (PCA) is applied to examine the stability of principal motion modes. The free energy landscape (FEL) is analyzed for consistency in the distribution of free energy states throughout the simulation. The radius of gyration (Rg) is used to assess the stability of the molecular compactness. Post-MD molecular mechanics/generalized Born surface area (MM/GBSA) calculations are performed to ensure consistent estimates of binding free energy. Finally, the solvent accessible surface area (SASA) is evaluated to determine the stability of solvent exposure of the molecular structure.

MDS for the 7 selected compounds was conducted for 100 ns. Out of 7 compounds only 3 compounds; STOCK1N-85097, STOCK1N-85998 & STOCK1N-86060, show good results and are discussed below. Complexes of Protein and ligand were generated, where **Complex-1** indicate 8EXV-STOCK1N-85097 complex, **Complex-2** indicates 8EXV-STOCK1N-85998 complex, **Complex-3** indicates 8EXV- STOCK1N-86060 complex, and **Complex-4** indicates 8EXV-Inavolisib (Co-crystalized Ligand) complex.

### Root mean square deviation (RMSD) of protein and lig fit prot

**Fig. 2:**
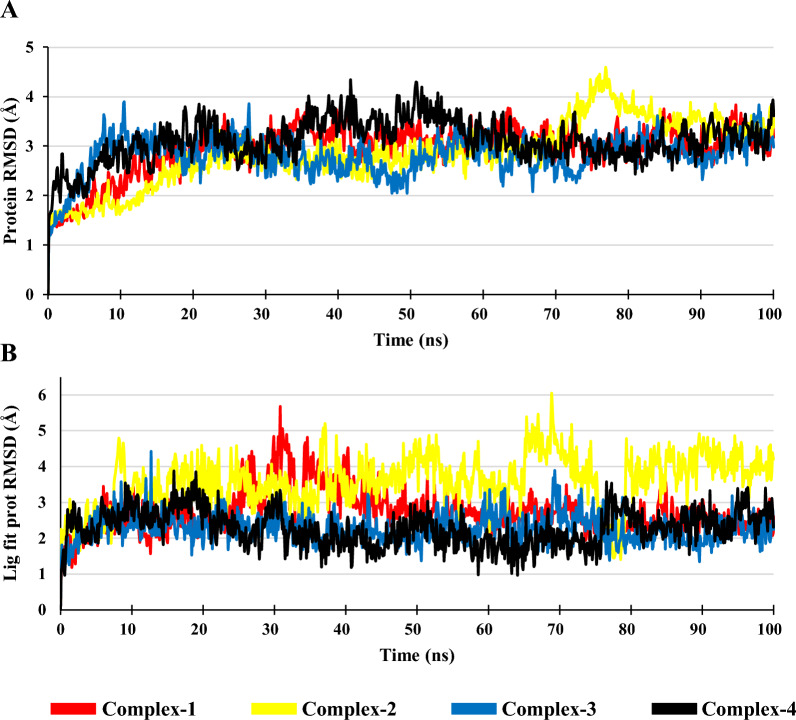
**A** Protein RMSD of the Complexes and **B** Lig fit prot RMSD of the Complexes

During the 100 ns simulation, the Protein RMSD of Complex-1, Complex-2, Complex-3 & Complex-4 was in the range of 1.22−3.841 Å, 1.318–4.597 Å, 1.122–3.902 Å and 1.464–4.347 Å, respectively, with an average RMSD of 2.95 Å, 2.88 Å, 2.81 Å and 3.12 Å, respectively. Protein RMSD of all the complexes are stable with slight increase was observed for Complex-2 at 70 ns to 78 ns, after which it becomes stable (Fig. 2A). Lig fit prot RMSD of Complex-1, Complex-2, Complex-3 & Complex-4 was in the range 0.997–5.673 Å, 1.403–6.048 Å, 1.247–4.427 Å, & 0.963–3.877 Å, respectively (Fig. 2B). Complex-3 and Complex-4 were stable through the simulation duration with Complex-1 and complex-2 showing some instability at 24 ns till 42 ns and 65 ns till 79 ns, respectively. As compared to other Complexes, the Complex-3 show similar protein and lig fit prot RMSD as Complex-4.

### Root mean square fluctuation (RMSF)

**Fig. 3:**
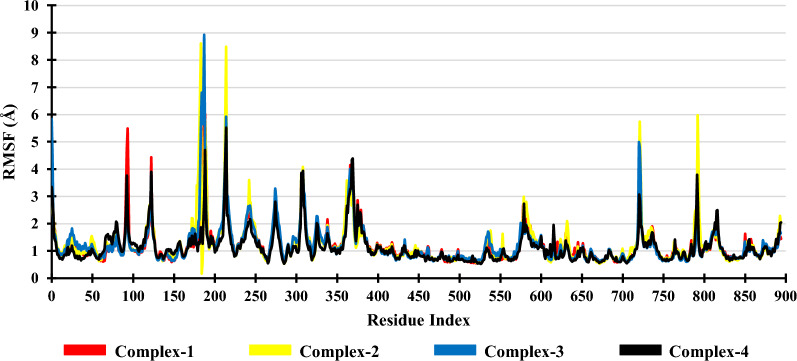
RMSF of the Complexes

RMSF detects the residues responsible for variations in complex structure and illustrates specific changes occurring along the protein chain. The main amino acid residues, namely, TRP780, TYP836, VAL851, ASP859 and ASP933, RMSF values were determined to identify the changes occurring during the simulations. In Complex-1, the main amino acids TRP780, TYP836, VAL851, ASP859 & ASP933 show RMSF value of 0.669 Å, 0.579 Å, 0.701 Å, 1.098 Å & 0.914 Å, respectively. In Complex-2, amino acids TRP780, TYP836, VAL851, ASP859 & ASP933 show RMSF value of 0.806 Å, 0.765 Å, 0.742 Å, 1.202 Å & 0.895 Å, respectively. In Complex-3, the RMSF value of key amino acids TRP780, TYP836, VAL851, ASP859 & ASP933 are 0.726 Å, 0.634 Å, 0.616 Å, 1.082 Å & 0.819 Å, respectively. In Complex-4, the RMSF value of key amino acids TRP780, TYP836, VAL851, ASP859 & ASP933 are 0.638 Å, 0.611 Å, 0.667 Å, 1.073 Å & 0.875 Å, respectively. The fluctuations observed in the key amino acids of the Complexes (1, 2 & 3) were similar to that of Complex-4 (Fig. 3).

### Ligand–protein complex interaction

**Fig. 4:**
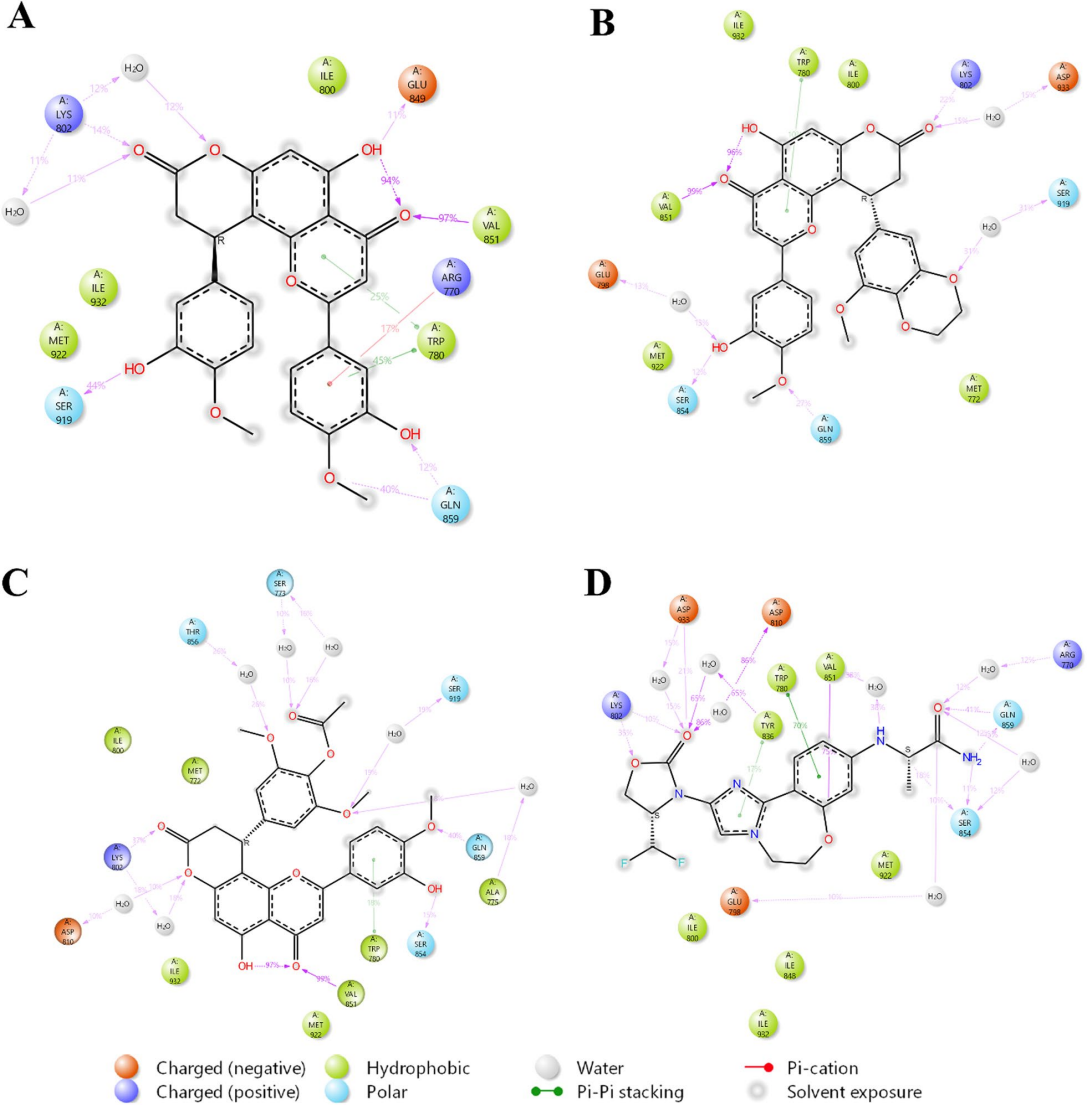
Ligand–Protein complex interaction after MDS – **A** Complex-1, **B** Complex-2, **C** Complex-3, and **D** Complex-4

Hydrogen bonds, π-π stacking, and π-cation stacking are essential for stabilizing protein–ligand interactions. Hydrogen bonds, formed between a hydrogen atom and electronegative atoms, improve binding strength, help maintain the ligand's correct position, and ensure specificity. π-π stacking, which involves interactions between aromatic rings, provides extra stability and enhances the binding affinity. π-cation stacking, where aromatic rings interact with positively charged cations, further stabilizes the complex and supports precise ligand placement. Together, these interactions reinforce the stability and effectiveness of the ligand–protein binding. In Complex-1, the ligand form Hydrogen bond with LYS802, GLU849, VAL851, GLN859 & SER919, water mediated H-bond with LYS802, π-cation stacking with ARG770 and π-π stacking with TRP780 (Fig. 4A). In Complex-2, ligand form Hydrogen bond with LYS802 VAL851, SER854, GLN859 & LYS802, water mediated H-bond with GLU798, SER919, ASP933 and π-π stacking with TRP780 (Fig. 4B). In Complex-3, ligand form Hydrogen bond with VAL851, SER854, GLN859 & LYS802, water mediated H-bond with SER773, ALA775, LYS802, ASP810, THR856 & SER919 and π-π stacking with TRP780 (Fig. 4C). In Complex-4, the ligand form Hydrogen bond with VAL851, SER854, GLN859, LYS802, and ASP933, water mediated H-bond with ARG770, GLU798, ASP810, TYR836, VAL851, SER854, & ASP933 and π-π stacking with TRP780 & TYR836 (Figure D). It is observed that all the ligands in the Complexes (1, 2 & 3) maintain an interaction similar to that of Complex-4.

### Protein–ligand contact timeline

**Fig. 5:**
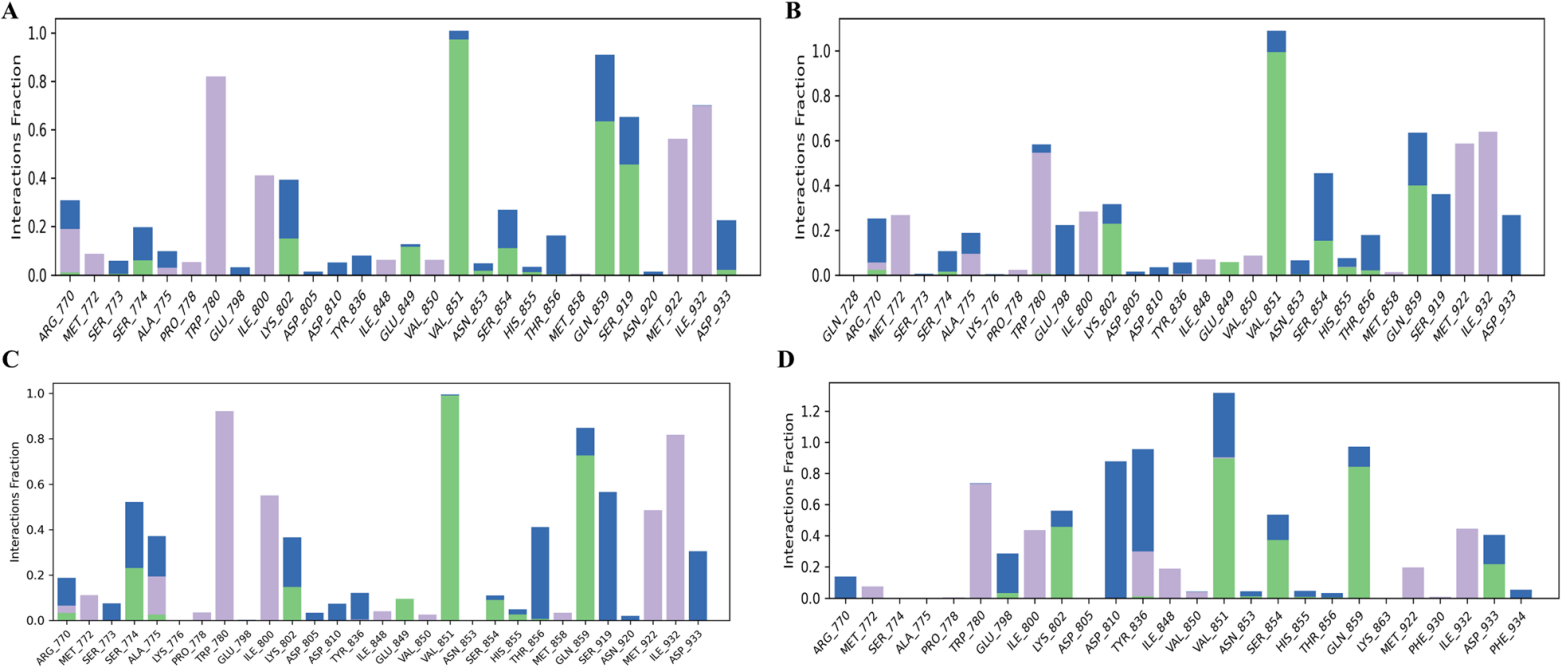
Ligand–Protein complex timeline – **A** Complex-1, **B** Complex-2, **C** Complex-3, and **D** Complex-4

**Fig. 6:**
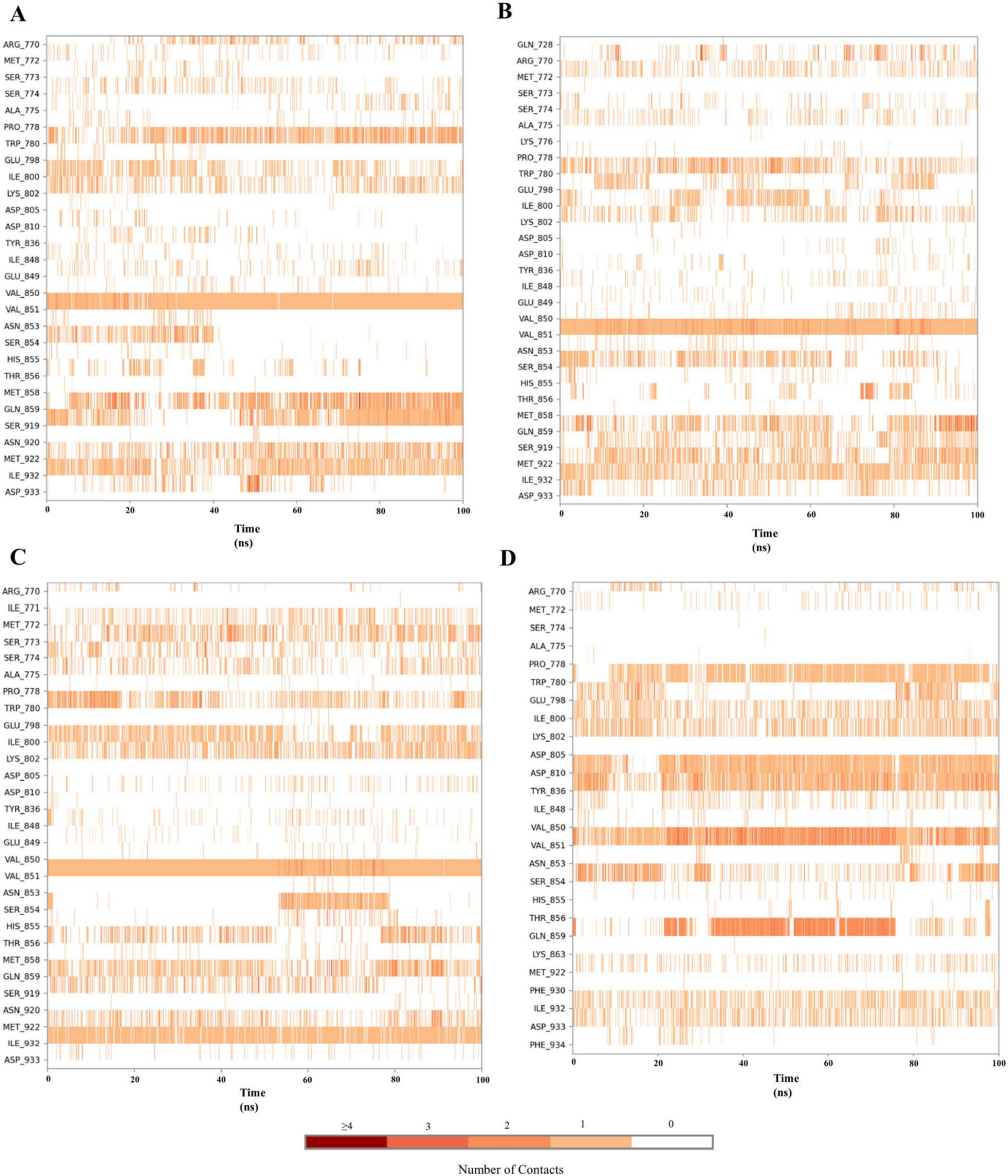
Ligand–Protein complex timeline – **A** Complex-1, **B** Complex-2, **C** Complex-3, and **D** Complex-4

The protein–ligand contact timeline illustrates the duration of interactions between the ligand and the amino acids of the protein during the simulation. If the interaction value exceeds 1, it indicates that multiple amino acids were involved in the interaction. A value of 0.3 signifies that the interaction was maintained for 30% of the simulated time. This timeline aids in understanding which amino acids interacted with the ligand and for how long during the simulation. In Complex-1, the amino acids ARG770, TRP780, ILE800, LYS802, VAL851, SER854, GLN859, SER919, MET922, ILE932 & ASP933 show interaction value of 0.299, 0.820, 0.412, 0.563, 1.009, 0.270, 0.910, 0.653, 0.562, 0.700 & 0.226, respectively. In Complex-2, the interaction value of amino acids ARG770, MET772, TRP780, ILE800, LYS802, VAL851, SER854. GLN859, SER919, MET922, ILE932 & ASP933 is 0.262, 0.268, 0.576, 0.222, 0.283, 0.316, 1.090, 0.455, 0.636, 0.360, 0.585, 0.638 & 0.268, respectively. In Complex-3 the interaction value of amino acids ARG770, SER774, ALA775, TRP780, ILE800, LYS802, VAL851, THR856, GLN859, SER919, MET922, ILE932 & ASP933 is 0.185, 0.517, 0.366, 0.917, 0.541, 0.360, 0.990, 0.405, 0.844, 0.561, 0.483, 0.815, & 0.205, respectively. In Complex-4, the amino acids TRP780, GLU798, ILE800, LYS802, ASP810, TYR836, VAL851, SER854, GLN859, ILE932 & ASP933 show interaction value of 0.735, 0.287, 0.437, 0.560, 0.878, 0.945, 1.312, 0.534, 0.971, 0.446 & 0.407, respectively. The interaction value of key amino acids of Complexes (1, 2 & 3) is similar to Complex-4. The ligand–protein contact histogram and timeline are shown in Figs. 5 and 6.

### Radius of gyration (Rg) and solvent accessible surface area (*SASA*)

**Fig. 7:**
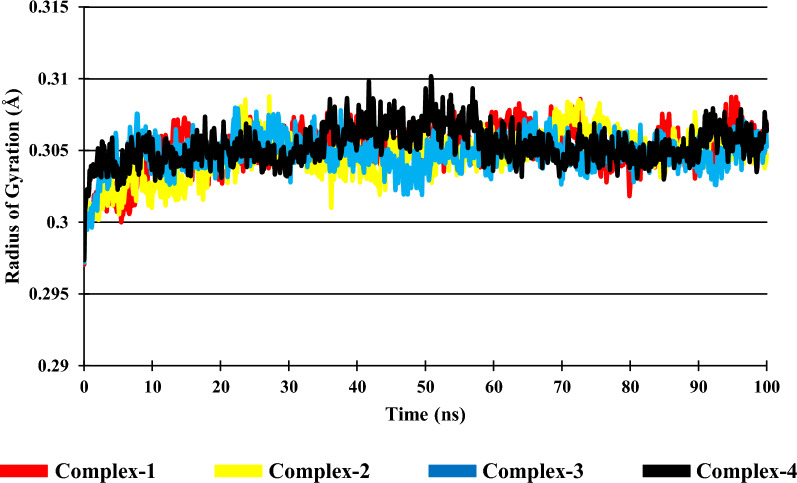
Radius of gyration of the complexes

The compactness of the Ligand–protein complex is evaluated by using the radius of gyration. The less the value of Rg, more the stability of folded protein which in turn indicates dynamic stability and compactness of the complex. The Complex-1 has an average Rg value of 0.3050 Å and has a range of 0.2970 Å to 0.3087 Å. In Complex-2, the average Rg is 0.3046 Å and has a range of 0.2977 Å to 0.3087 Å. The Complex-3 has an average Rg value of 0.3047 Å and has a range of 0.2971 Å to 0.3080 Å. The Complex-4 has an average Rg value of 0.3053 and has a range of 0.2973 Å to 0.3101 Å. The average Rg and the range of the Complexes (1, 2 & 3) is found to be marginally smaller than the Complex-4 and shown in Fig. 7.

**Fig. 8:**
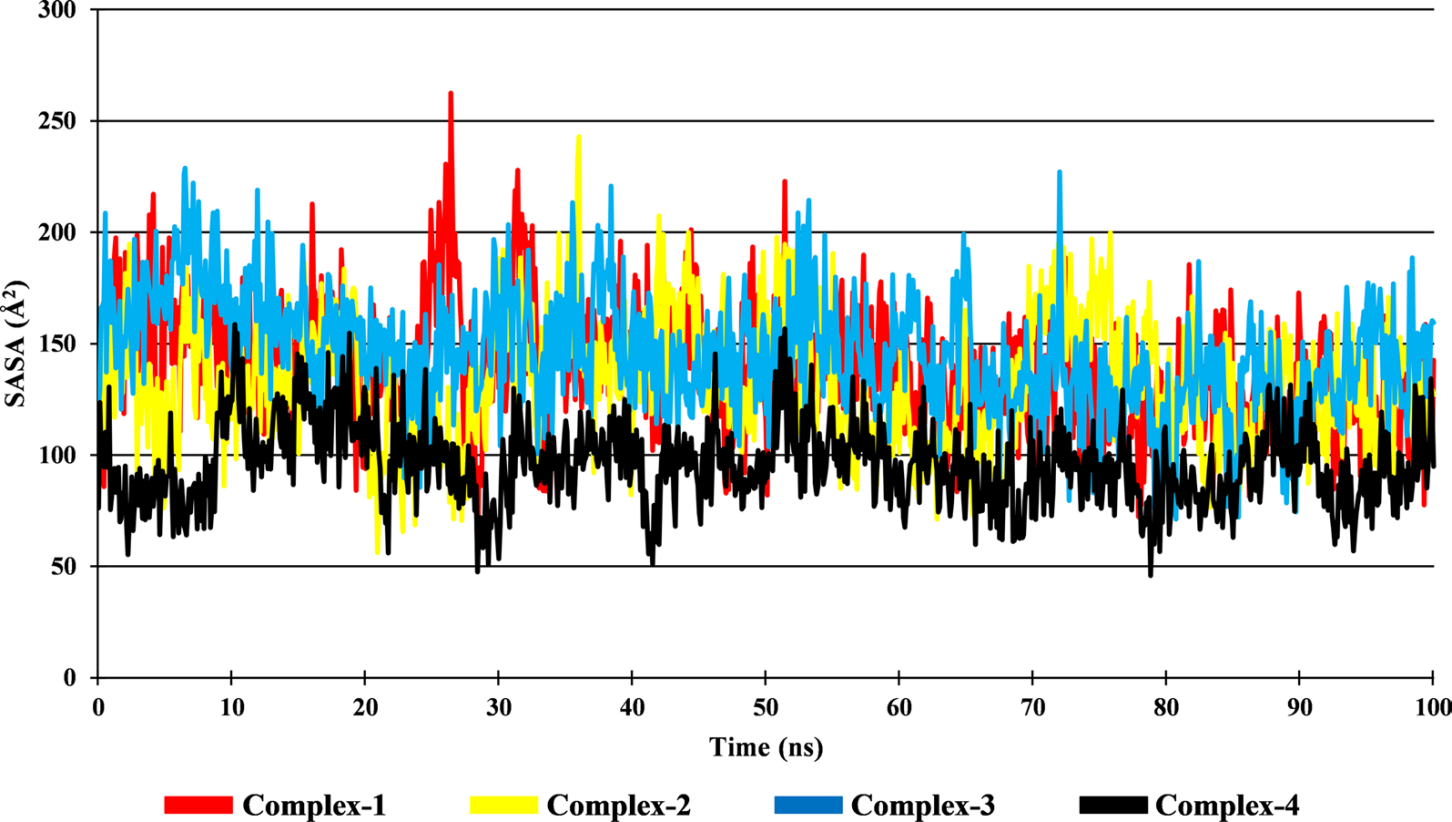
Solvent accessible surface area of the complexes

Solvent Accessible Surface Area (SASA) gives an idea about the area of ligand accessible to the solvent in Ligand–Protein complex. The SASA value of Complex-1, Complex-2, Complex-3, and Complex-4 is in the range of 52.04 Å^2^ to 262.55 Å^2^, 56.23 Å^2^ to 242.87 Å^2^, 68.70 Å^2^ to 228.78 Å^2^ and 45.87 Å^2^ to 158.74 Å^2^, respectively. The average SASA value for Complex-1, Complex-2, Complex-3, and Complex-4 is 138.24 Å^2^, 132.97 Å^2^, 145.14 Å^2^ and 95.66 Å^2^, respectively (Fig. 8). It was observe that Complex-4 has lower SASA value as compared to other Complexes (1, 2 & 3).

### Hydrogen bond (H-Bond) analysis

**Fig. 9:**
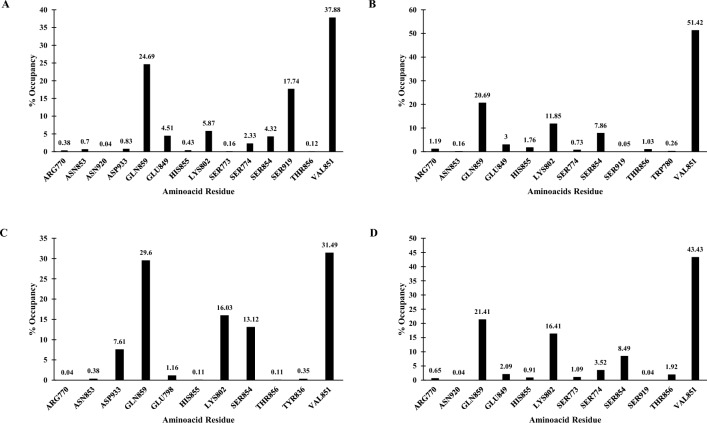
Hydrogen bond histogram – **A** Complex-1, **B** Complex-2, **C** Complex-3, and **D** Complex-4

Hydrogen bond plays a crucial role in the stability of Lignd-Protein complex. Its analysis helps in understanding the interactions between the ligand and the protein's amino acids and the rearrangements occur in the protein binding site upon ligand interaction. In Complex-1, 2571 H-Bond was formed between the ligand and the protein with VAL851, GLN859 and SER919 forming the majority of bond with 37.88%, 24.69% and 17.74%, respectively (Fig. 9A). In Complex-2, 1933 H-bond was formed with the majority of bond formed with LYS802 (11.85%), VAL851 (51.42%) and GLN859 (20.69%) (Fig. 9B). In Complex-3, a total of 2298 H-bond was formed with LYS802 (16.41%), VAL851 (43.43%) and GLN859 (21.41%) forming the majority of the bond (Fig. 9C). In Complex-4, 2851 H-bond was formed with majority of bond formed by LYS802 (16.03%), VAL851 (31.49%), SER854 (13.12%) and GLN859 (29.60%) (Fig. 9D).

### Principal component analysis (PCA)

**Fig. 10:**
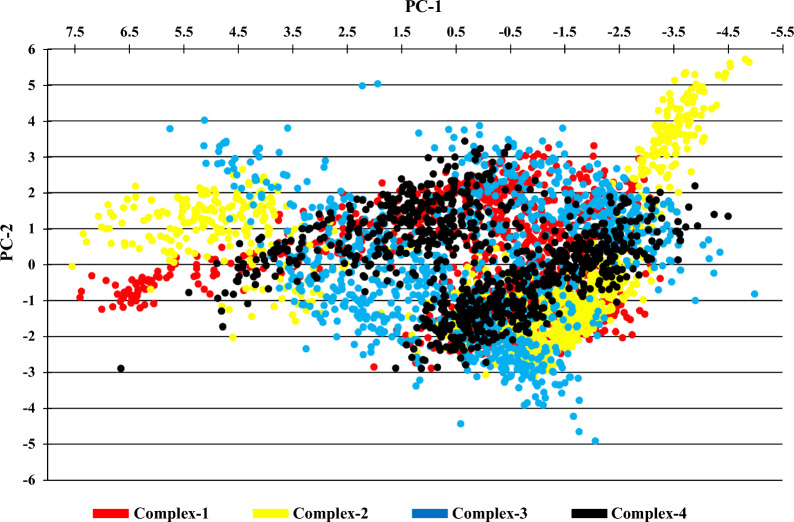
Scatter plot of PC1 vs PC2 of Complexes

Principal Component study (PCA) is a very useful method for simplifying the complexity and recognizing crucial motions during the ligand–protein binding. Based on the trajectory a matrix is generated which excludes translational and rotational movements. The eigenvectors, eigenvalues, and their projections onto the first two Principal Components (PCs) were then calculated using the essential dynamics procedure. The eigenvalues and eigenvectors were obtained by diagonalizing the matrix; the eigenvalues were found to be the associated eigenvectors' magnitude. The multidimensional space is described by the matrix of eigenvectors, which also gives details on the atoms' displacements along each direction in the protein. Geo-Measures: A PyMOL plugin was used for generating the PCs [20]. In the Complex-1, the eigenvalues of PC-1 and PC-2 were in the range of − 3.246 nm to 7.404 nm and − 2.873 nm to 3.313 nm, respectively. The eigenvalues for PC-1 and PC-2 for Complex-2 is in the range of − 4.876 to 7.549 nm and − 3.101 to 5.718 nm, respectively. For Complex-3, the eigenvalues of PC-1 and PC-2 is in the range of − 4.981 to 5.758 nm and − 4.904 to 5.041 nm, respectively. For Complex-4, the eigenvalues of PC-1 and PC-2 is in the range of − 4.494 to 6.655 nm and − 2.884 to 3.442 nm, respectively. Compared to Complex-2, Complex-1 and Complex-3 shows a very steady cluster and took up the least amount of phase space. The Complex-4 (Co-crystalized ligand) shows better values and clusters as compared to all the other Complexes. The Scatter plot of PC-1 and PC-2 of the Complexes are shown in Fig. 10 and Cross-correlation of the Complexes is shown in Supplementary file Figure S1.

### Free energy landscape (FEL)

**Fig. 11:**
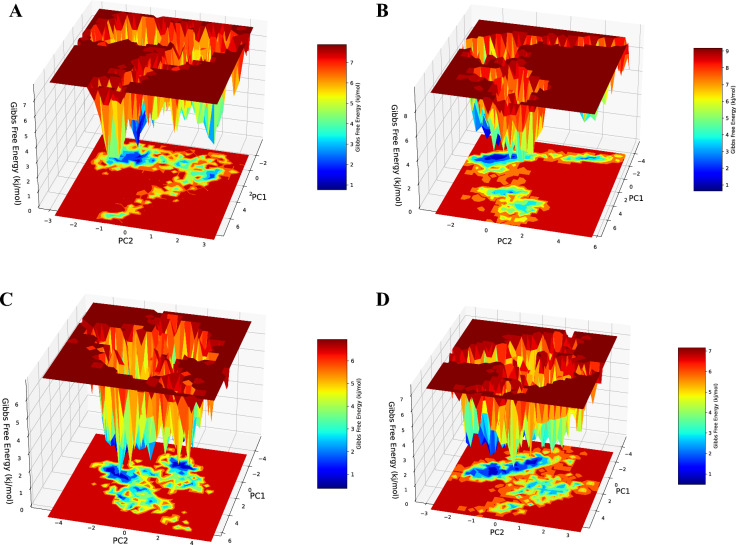
3D Free Energy Landscape – **A** Complex-1, **B** Complex-2, **C** Complex-3, and **D** Complex-4

Energy analysis was performed using conformations produced by MD simulations. The protein free energy landscape (FEL), which provides information on structural changes and stability, may be used to analyze the energy distribution of a protein in a range of variables, including principal components (PCs). Geo-Measures: A PyMOL plugin was used for generating the FEL for the Complexes using the PC-1 and PC-2 eigenvalues which gives details about the protein's changes in conformation and stability. The Dark blue region (Less energy) indicates favorable conformation of protein and Red region indicates higher energy reflecting the unfavorable conformations. The Gibbs free energy of Complex-1, Complex-2, Complex-3, and Complex-4 is in the range of 1.3 to 7.87 kj/mol, 0 to 9.17 kj/mol, 0 to 6.94 kj/mol and 0 to 7.16 kj/mol, respectively. Out of the Complexes (1, 2 & 3), Complex-3 shows less Gibbs free energy range than Complex-4 (Fig. 11).

### Post MDs binding free energy (MMGBSA dG bind) and total energy

**Fig. 12:**
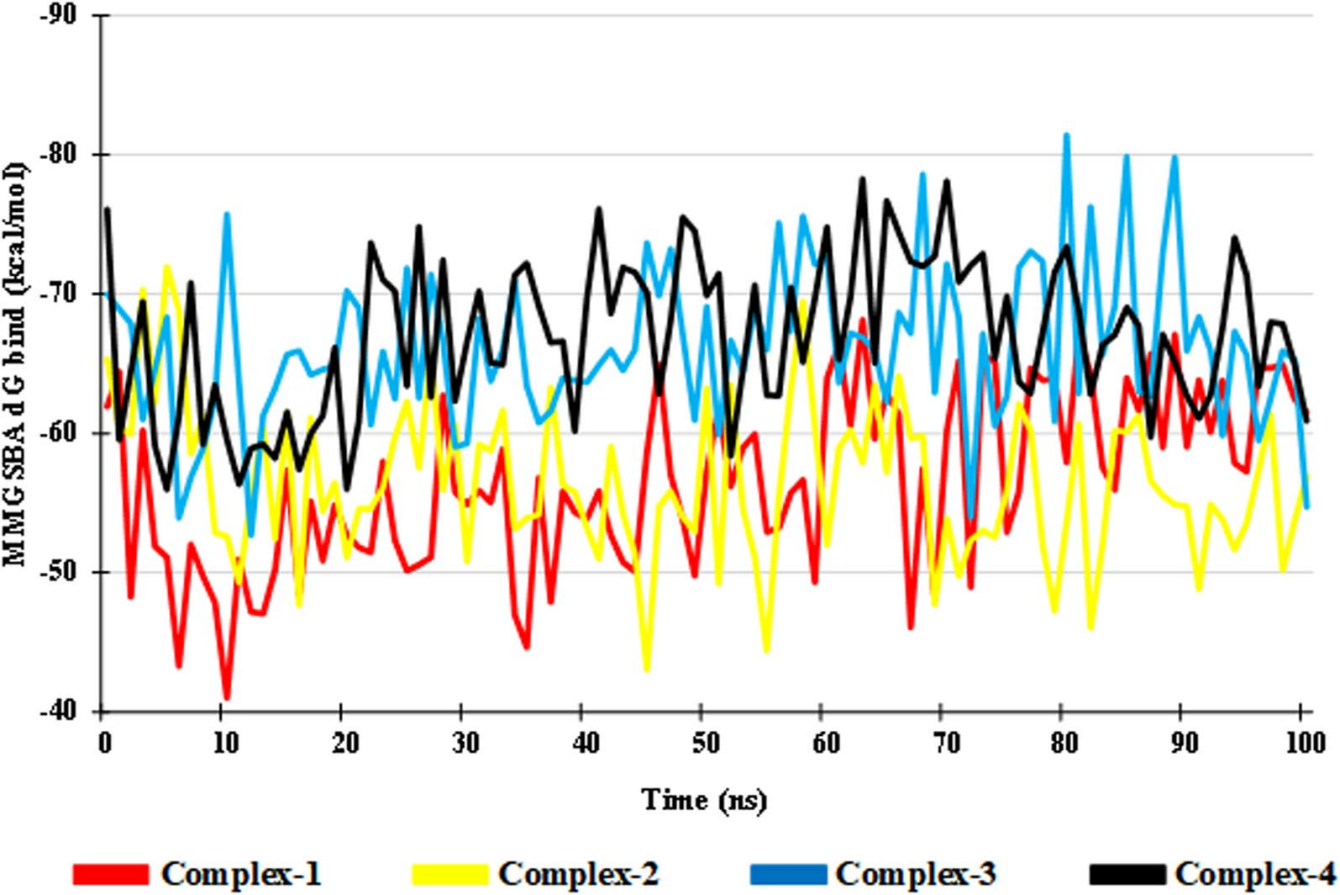
Post MDs Binding Free energy of the Complexes

Post MDs Binding Free energy gives an idea about the stability of ligand–protein complex. The lower the value more the stability. The Complex-1, Complex-2, Complex-3 and Complex-4 show an average MMGBSA dG bind value of − 56.55 kcal/mol, − 56.53 kcal/mol, − 66.18 kcal/mol and − 66.93 kcal/mol, respectively. The Complex-1, Complex-2, Complex-3, and Complex-4 MMGBSA dg bind value lie in the range of − 68.15 to − 41.04 kcal/mol, − 71.94 to − 43.04 kcal/mol, − 81.38 to − 52.70 kcal/mol and − 78.26 to − 55.98 kcal/mol, respectively. Out of the Complexes (1, 2 & 3), the Complex-3 shows a similar average MMGBSA dg bind value and lower energy range as compared to the Compex-4 (Fig. 12).

**Fig. 13:**
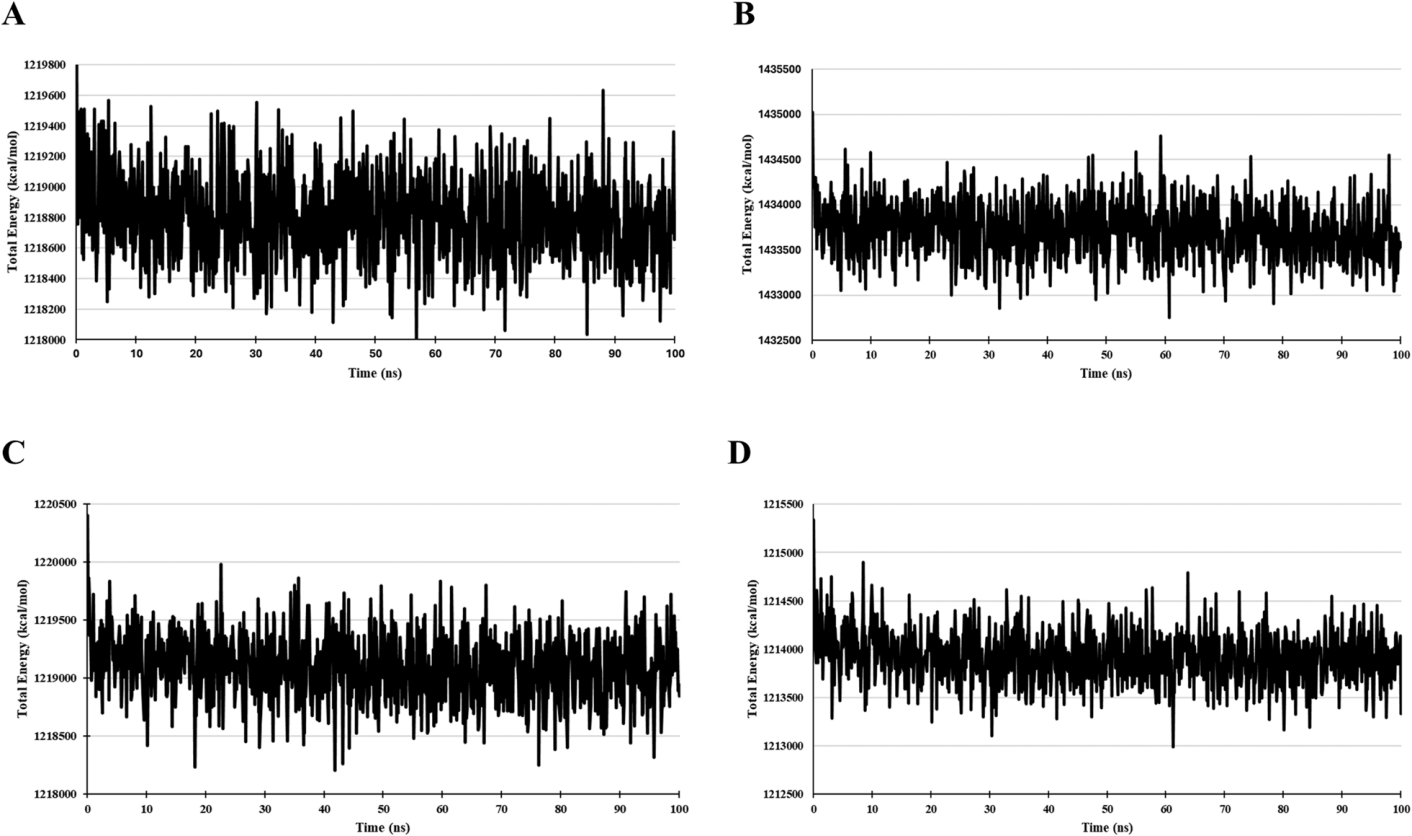
Total Energy of – **A** Complex-1, **B** Complex-2, **C** Complex-3, and **D** Complex-4

The Ligand–protein complex total energy is shown in Fig. 13. The average total energy of the Complex-1, Complex-2, Complex-3 and Complex-4 is 1,218,800 kcal/mol, 1,433,712 kcal/mol, 1,219,093 kcal/mol and 1,213,924 kcal/mol, respectively. The lower the total energy of Ligand–protein complex higher its stability, Complex-1 and Complex-3 show similar energy range to that of Complex-4 signifying the stability of the protein ligand complex. The total energy of Complex-2 is found to be higher than all the other Complexes.

## Conclusion

PI3K-α belongs to the Class-1 subdivision of PI3K, and its mutation is responsible for about 29% of breast cancers and roughly 40% of HR + /HER2- metastatic breast cancers. This study employed computational techniques to identify natural compounds: isoform and mutant specific PI3K-α inhibitors. The e-Pharmacophore model was used to generate a hypothesis using Inavolisib, which is under clinical trial Phase-3 for indication of HR + , HER2-negative, PIK3CA mutated mBC post CDK4/6i therapy (PDB: 8EXV). Phase screening, ligand docking, induced-fit docking, and ADMET analysis, Seven compounds were selected for molecular dynamics simulation analysis. Out of those seven compounds, only three compounds, namely STOCK1N-85097, STOCK1N-85998, and STOCK1N-86060, show good RMSD, RMSF, Rg, SASA, PCA, FEL, and Total energy. It was observed that the Ligands made interaction with amino acids residue ARG770, ASP (810 & 933), GLN859, GLU798, LYS802, SER854, TRP780, and VAL851 of the PI3K-α protein, which is similar to the Co-crystalized Ligand (Inavolisib). Therefore, it can be predicted that the compounds will follow similar mechanism of action to inhibit PI3K–α protein. Out of three selected compounds, STOCK1N-86060 showed better results, and its values were significantly similar or better. Based on the present research, compound STOCK1N-86060 can be proposed as a potential inhibitor of PI3K-α, which should be validated using proper in-vitro and in-vivo models.

## Supplementary Information

Supplementary Material 1.

## Data Availability

The datasets used and/or analyzed during the current study are available from the corresponding author on reasonable request.

## Abbreviations

ARG: Arginine
ASN: Asparagine
FEL: Free Energy Landscape
GLN: Glutamine
HIS: Histidine
HTVS: High-throughput virtual screening
IFD: Induced fit docking
ILE: Isoleucine
LEU: Leucine
MDS: Molecular dynamic simulation
PCA: Principal component analysis
PI3K-α: Phosphatidylinositol 3-kinase catalytic 110-KD alpha
RMSD: Root mean square deviation
ROS: Reactive oxygen species
SASA: Solvent accessible surface area
Rg: Radius of Gyration
RMSF: Root mean square fluctuation
SER: Serine
SP: Standard precision
TYR: Tyrosine
VAL: Valine
XP: Extra precision
