# Supplementary material for: Identification of phytomolecules as isoform and mutation specific PI3K-α inhibitor for protection against breast cancer using e-pharmacophore modeling and molecular dynamics simulations
Source: BMC Chem. 2024 Dec 18;18(1):241. doi: 10.1186/s13065-024-01317-w (PMC11657504; doi:10.1186/s13065-024-01317-w)
Supplement: Supplementary file 1 — Supplementary Material 1. [file 13065_2024_1317_MOESM1_ESM.docx]

Supporting Information

**Identification of Phytomolecules as Isoform and Mutation specific PI3K-α inhibitor for protection against Breast Cancer using e-Pharmacophore modeling and Molecular dynamics simulations.**

**Authors:** Ajay Mili ^a^, Sumit Birangal ^b^, Jyothi Giridhar ^b^, Krishnadas Nandakumar ^c^, Richard Lobo ^a,^ *

*^a^Department of Pharmacognosy, Manipal College of Pharmaceutical Sciences, Manipal Academy of Higher Education, Manipal, Karnataka-576104, India.*

*^b^Department of Pharmaceutical Chemistry, Manipal College of Pharmaceutical Sciences, Manipal Academy of Higher Education, Manipal, Karnataka-576104, India.*

*^c^Department of Pharmacology, Manipal College of Pharmaceutical Sciences, Manipal Academy of Higher Education, Manipal, Karnataka-576104, India.*

**** Corresponding author***

*Dr. Richard Lobo*

Email id: richard.lobo@manipal.edu

Contact no: +91-9448104090

Table of Contents

| **Page no** | **Information** |
| --- | --- |
| S3-S5 | Table S1: Active set |
| S6-S11 | Table S2: 2D interaction image of Ligand and 25 selected Compounds |
| S12-S17 | Table S3: IFD score and 3D interaction image of the 25 selected Compounds |
| S18 | Figure S1: Cross-correlation of the Complexes. |

**Table no S1: Actives Set**

| **Sl no** | PubChem ID | **Structure** | **IUPAC name** | **Ki (nM)** |
| --- | --- | --- | --- | --- |
|  | 58204997 |  | (S)-2-((2-(1-isopropyl-1H-1,2,4-triazol-5-yl)-5,6-dihydrobenzo[f]imidazo[1,2-d][1,4]oxazepin-9-yl)oxy)propanamide | 0.346 |
|  | 58205016 |  | (S)-2-((2-(1-(2,2,2-trifluoroethyl)-1H-1,2,4-triazol-5-yl)-5,6-dihydrobenzo[f]imidazo[1,2-d][1,4]oxazepin-9-yl)oxy)propanamide | 0.188 |
|  | 168297753 |  | (S)-2-((2-(3-(hydroxymethyl)-1-(2,2,2-trifluoroethyl)-1H-1,2,4-triazol-5-yl)-5,6-dihydrobenzo[f]imidazo[1,2-d][1,4]oxazepin-9-yl)oxy)propanamide | 0.062 |
|  | 168297494 |  | (S)-1-(2-(3-amino-1-isopropyl-1H-1,2,4-triazol-5-yl)-5,6-dihydrobenzo[f]imidazo[1,2-d][1,4]oxazepin-9-yl)pyrrolidine-2-carboxamide | 0.026 |
|  | 168282983 |  | (S)-1-(2-(5,5-dimethyl-2,4-dioxoimidazolidin-1-yl)-5,6-dihydrobenzo[f]imidazo[1,2-d][1,4]oxazepin-9-yl)pyrrolidine-2-carboxamide | 0.681 |
|  | 168272875 |  | (2S)-1-[2-(3-oxo-1,4-oxazepan-4-yl)-5,6-dihydroimidazo[1,2-d][1,4]benzoxazepin-9-yl]pyrrolidine-2-carboxamide | 0.32 |
|  | 165430683 |  | (2S)-1-[2-[(4R)-2-oxo-4-propan-2-yl-1,3-oxazolidin-3-yl]-5,6-dihydroimidazo[1,2-d][1,4]benzoxazepin-9-yl]pyrrolidine-2-carboxamide | 0.042 |
|  | 124169471 |  | (2S)-1-[2-[(4S)-2-oxo-4-(trifluoromethyl)-1,3-oxazolidin-3-yl]-5,6-dihydroimidazo[1,2-d][1,4]benzoxazepin-9-yl]pyrrolidine-2-carboxamide | 0.15 |
|  | 124169052 |  | (2S)-1-[2-[(4S)-4-(difluoromethyl)-2-oxo-1,3-oxazolidin-3-yl]-5,6-dihydroimidazo[1,2-d][1,4]benzoxazepin-9-yl]pyrrolidine-2-carboxamide | 0.053 |
|  | 124169085 |  | (2S)-1-[2-[(4S)-4-(fluoromethyl)-2-oxo-1,3-oxazolidin-3-yl]-5,6-dihydroimidazo[1,2-d][1,4]benzoxazepin-9-yl]pyrrolidine-2-carboxamide | 0.043 |
|  | 168274763 |  | (2S,3R)-3-methoxy-1-[2-[(4S)-2-oxo-4-(trifluoromethyl)-1,3-oxazolidin-3-yl]-5,6-dihydroimidazo[1,2-d][1,4]benzoxazepin-9-yl]pyrrolidine-2-carboxamide | 0.097 |
|  | 168295138 |  | (2S)-2-[[2-[(4S)-2-oxo-4-(trifluoromethyl)-1,3-oxazolidin-3-yl]-5,6-dihydroimidazo[1,2-d][1,4]benzoxazepin-9-yl]amino]propanamide | 0.095 |
|  | 165430682 |  | (2S)-2-cyclopropyl-2-[[2-[(4S)-2-oxo-4-(trifluoromethyl)-1,3-oxazolidin-3-yl]-5,6-dihydroimidazo[1,2-d][1,4]benzoxazepin-9-yl]amino]acetamide | 0.1 |
|  | 124173720 |  | (2S)-2-[[2-[(4S)-4-(difluoromethyl)-2-oxo-1,3-oxazolidin-3-yl]-5,6-dihydroimidazo[1,2-d][1,4]benzoxazepin-9-yl]amino]propanamide | 0.034 |
|  | 124173719 |  | (2S)-2-cyclopropyl-2-[[2-[(4S)-4-(difluoromethyl)-2-oxo-1,3-oxazolidin-3-yl]-5,6-dihydroimidazo[1,2-d][1,4]benzoxazepin-9-yl]amino]acetamide | 0.06 |

**Table no S2: 2D interaction image of Ligand and 25 selected Compounds**

| **Sl no** | **Compound id** | **2D interaction image** |
| --- | --- | --- |
|  | Inavolisib | 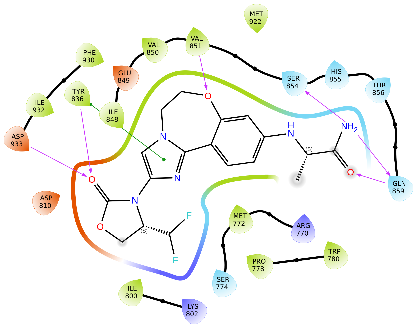 |
|  | STOCK1N-113116 | 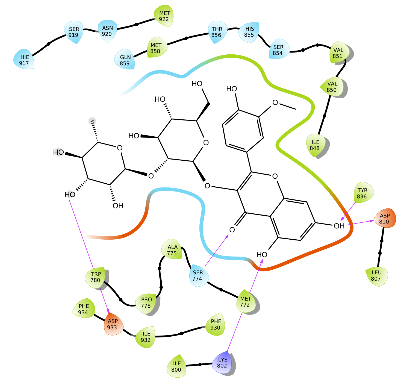 |
|  | STOCK1N-81073 | 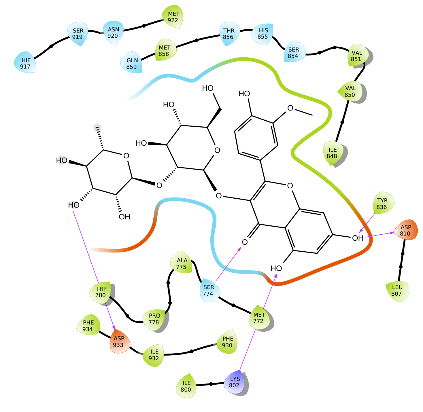 |
|  | STOCK1N-51145 | 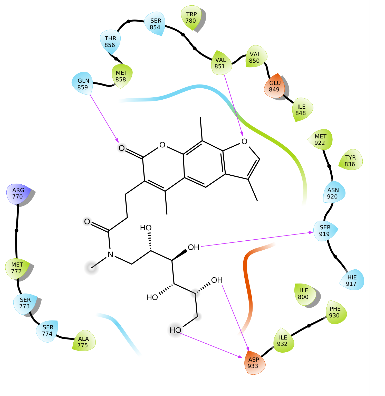 |
|  | STOCK1N-59405 | 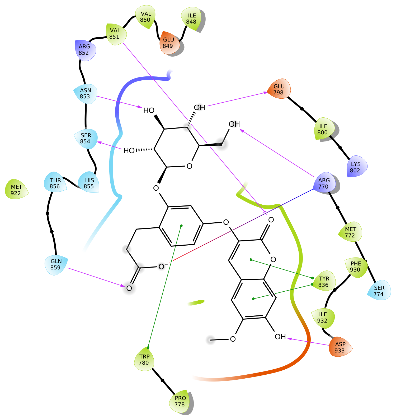 |
|  | STOCK1N-84648 | 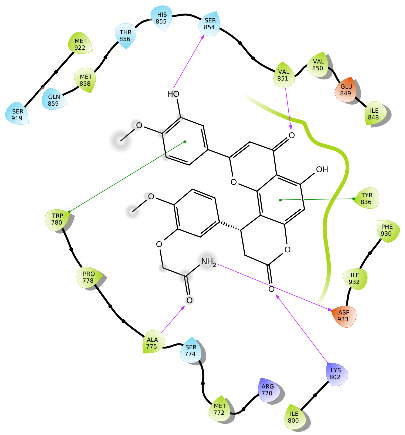 |
|  | STOCK1N-46158 | 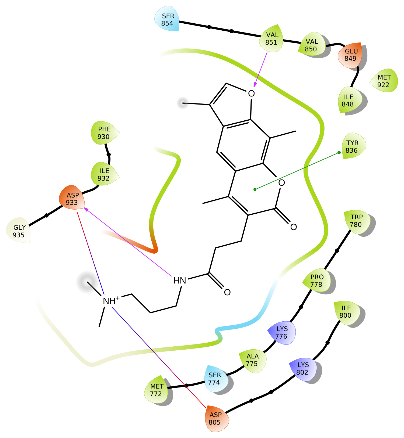 |
|  | STOCK1N-85097 | 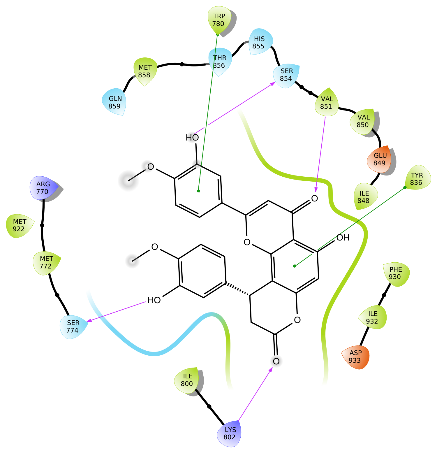 |
|  | STOCK1N-84443 | 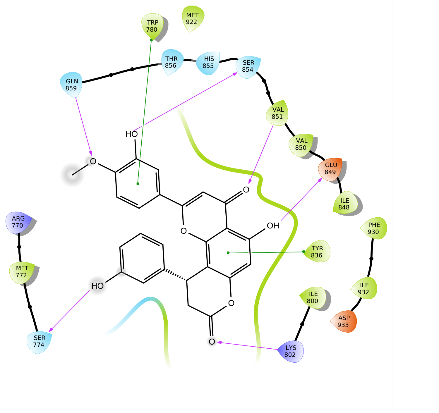 |
|  | STOCK1N-64228 | 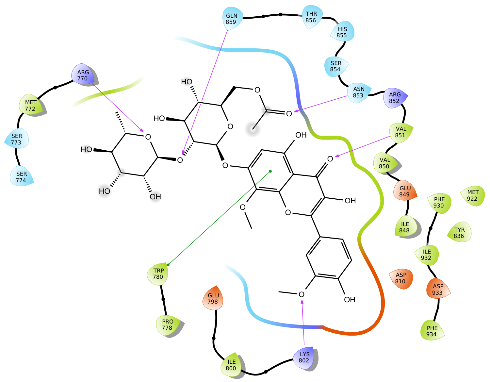 |
|  | STOCK1N-84098 | 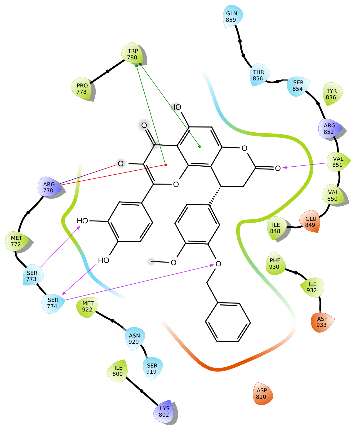 |
|  | STOCK1N-85873 | 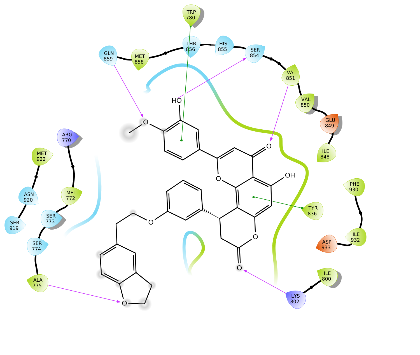 |
|  | STOCK1N-92951 | 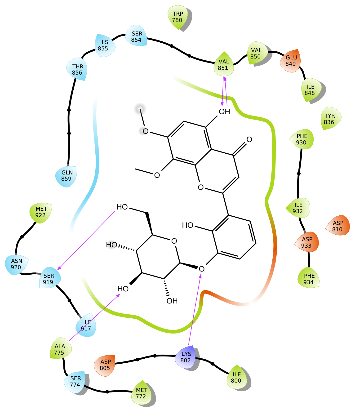 |
|  | STOCK1N-92949 | 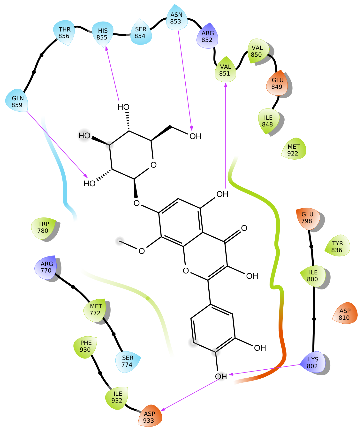 |
|  | STOCK1N-40367 | 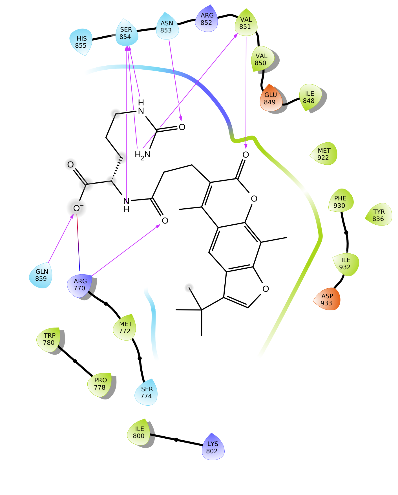 |
|  | STOCK1N-85433 | 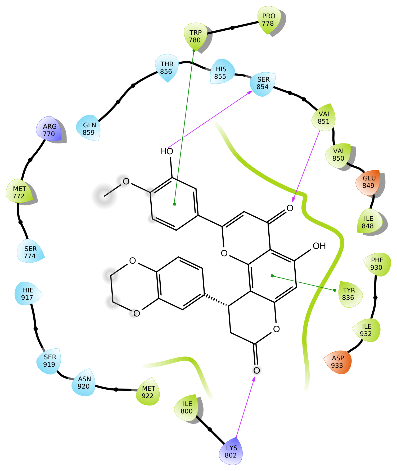 |
|  | STOCK1N-1935 | 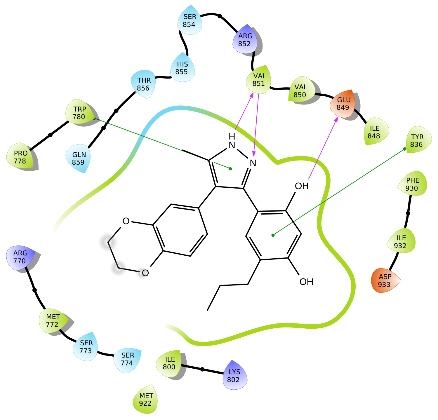 |
|  | STOCK1N-81980 | 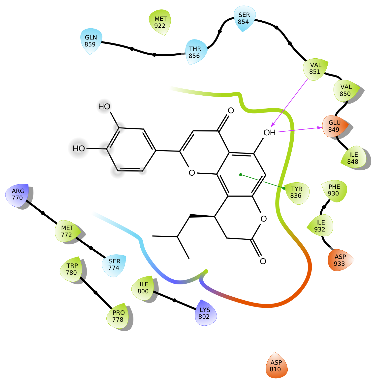 |
|  | STOCK1N-85851 | 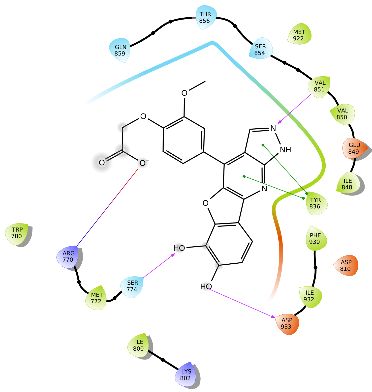 |
|  | STOCK1N-84154 | 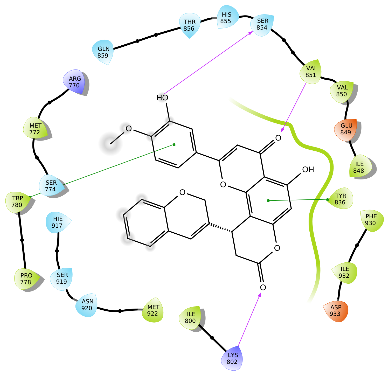 |
|  | STOCK1N-85998 | 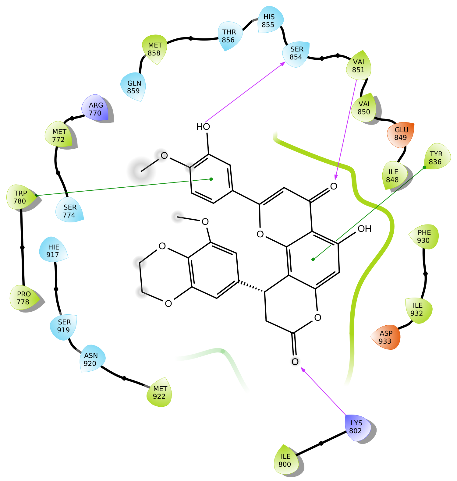 |
|  | STOCK1N-86351 | 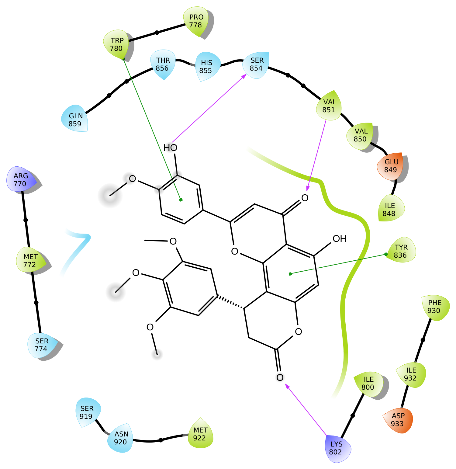 |
|  | STOCK1N-88743 | 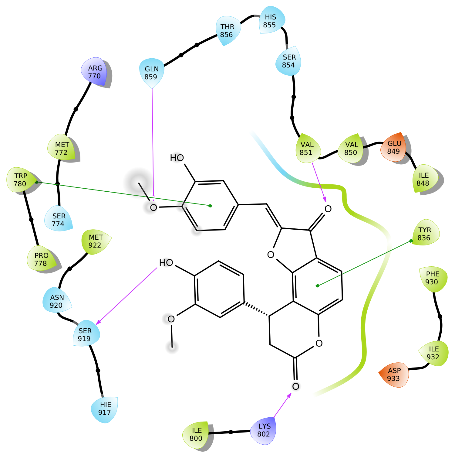 |
|  | STOCK1N-42002 | 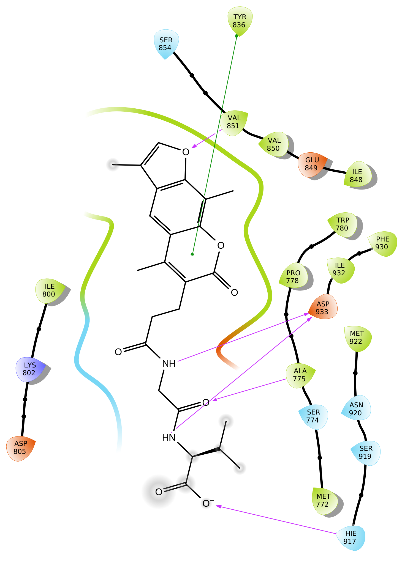 |
|  | STOCK1N-86060 | 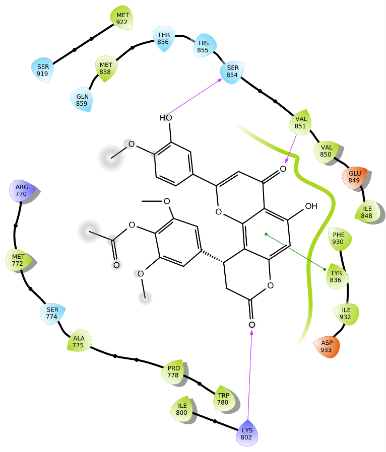 |
|  | STOCK1N-83010 | 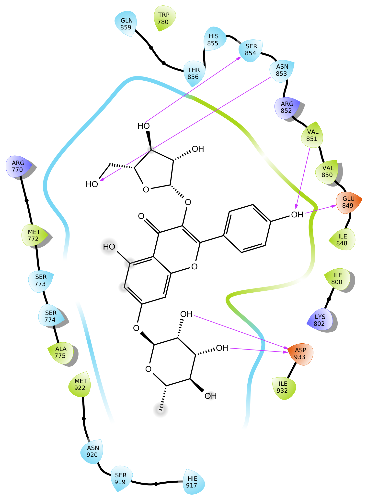 |

**Table S3: IFD score and 3D interaction image of the 25 selected Compounds**

| **Sl no** | **Compound** | **IFD score (kcal/mol)** | **3D structure of Best pose** |
| --- | --- | --- | --- |
|  | STOCK1N-113116 | -1924.09 | 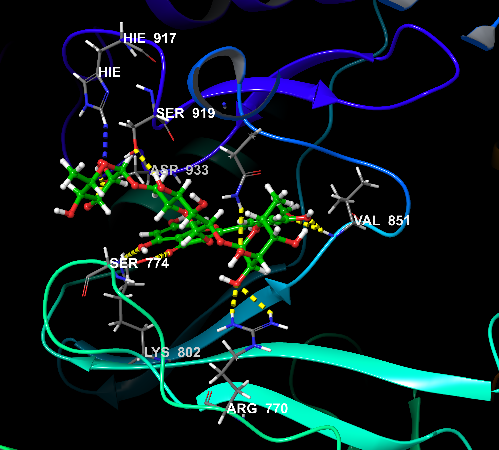 |
|  | STOCK1N-81073 | -1925.56 | 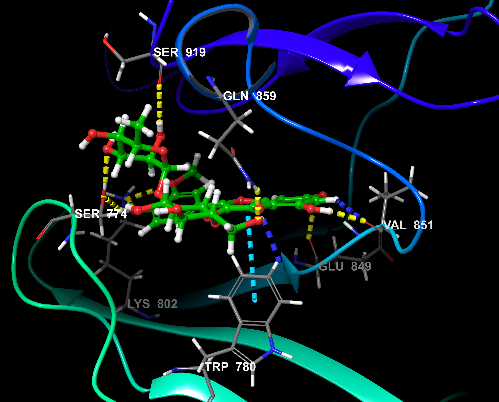 |
|  | STOCK1N-51145 | -1916.56 | 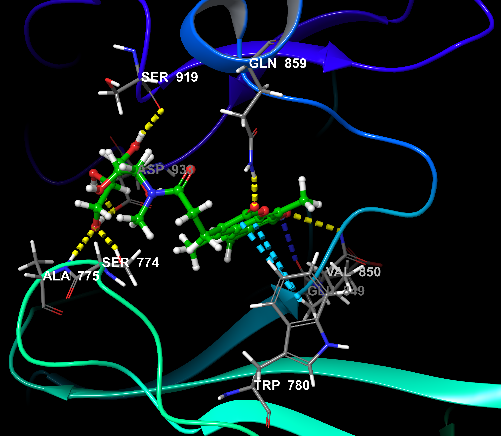 |
|  | STOCK1N-59405 | -1987.21 | 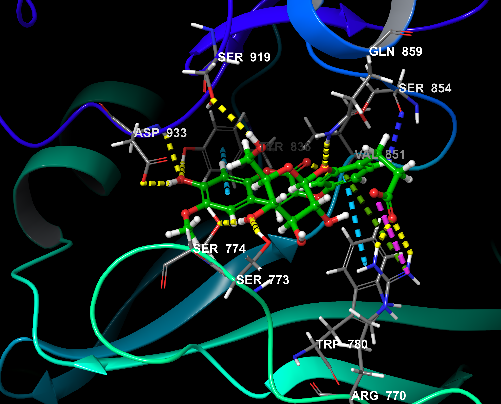 |
|  | STOCK1N-84648 | -1929.37 | 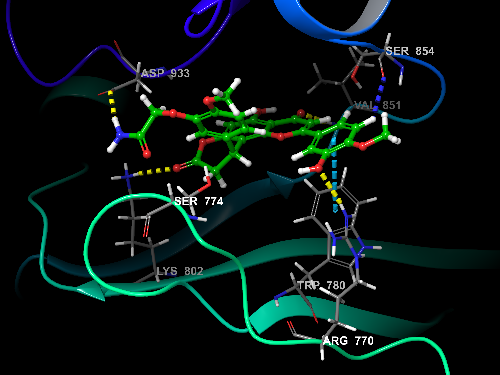 |
|  | STOCK1N-46158 | -1915.39 | 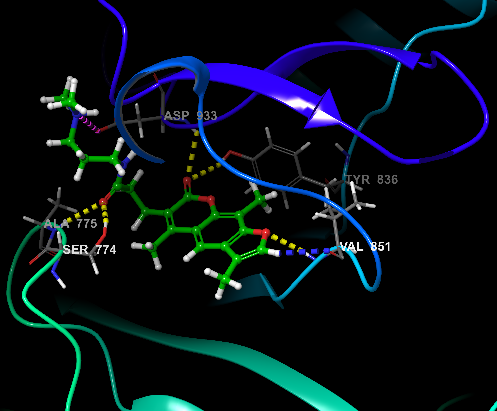 |
|  | STOCK1N-85097 | -1927.92 | 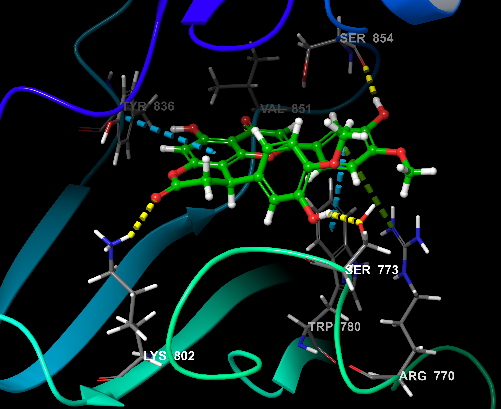 |
|  | STOCK1N-84443 | -1928.66 | 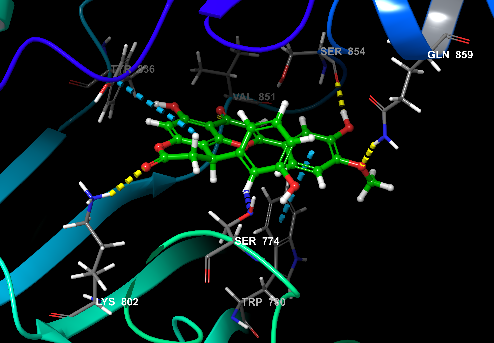 |
|  | STOCK1N-64228 | -1918.18 | 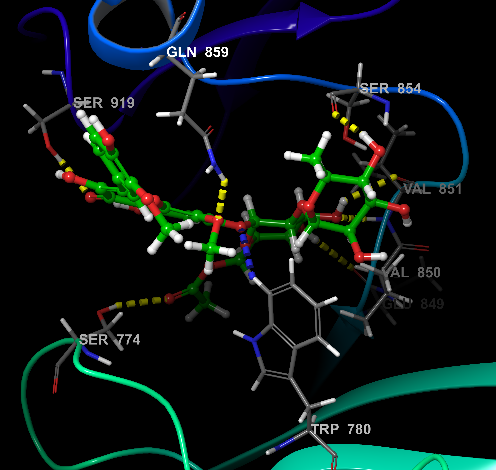 |
|  | STOCK1N-84098 | -1922.98 | 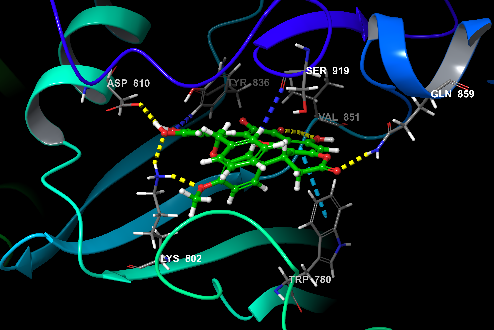 |
|  | STOCK1N-85873 | -1927.31 | 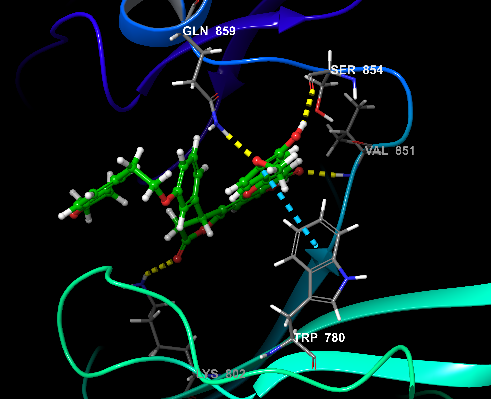 |
|  | STOCK1N-92951 | -1920.80 | 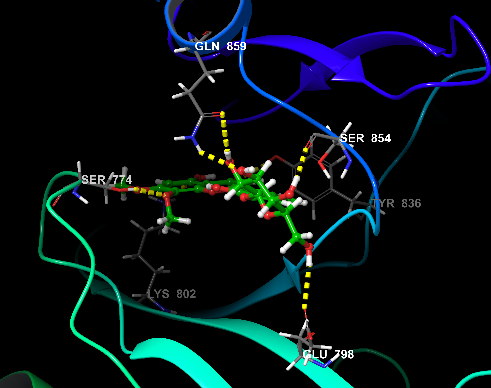 |
|  | STOCK1N-92949 | -1917.19 | 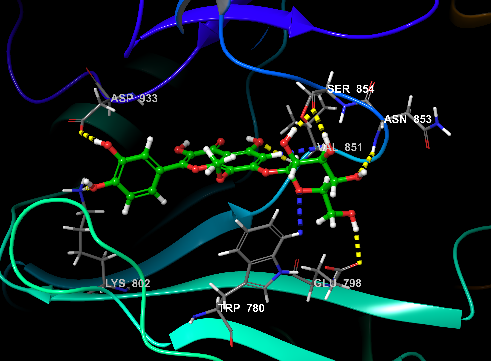 |
|  | STOCK1N-40367 | -1921.24 | 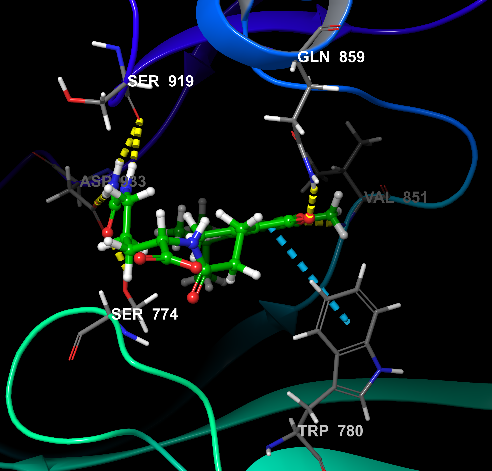 |
|  | STOCK1N-85433 | -1927.41 | 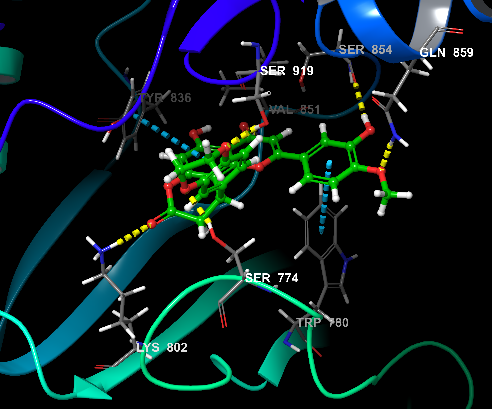 |
|  | STOCK1N-1935 | -1916.43 | 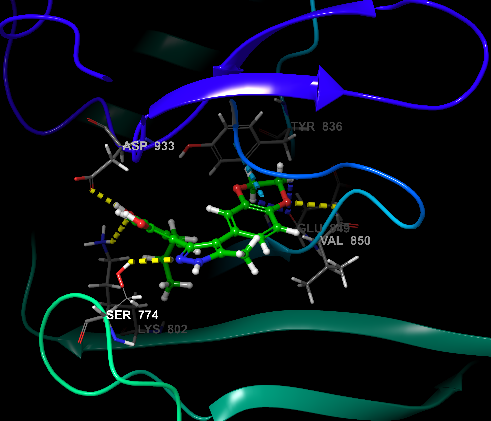 |
|  | STOCK1N-81980 | -1926.11 | 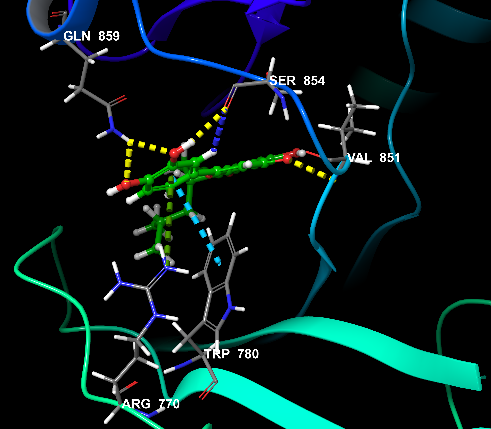 |
|  | STOCK1N-85851 | -1917.63 | 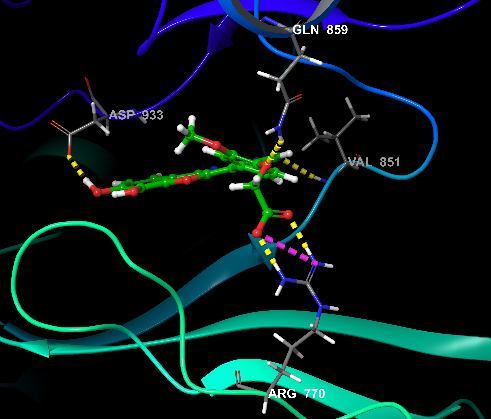 |
|  | STOCK1N-84154 | -1929.87 | 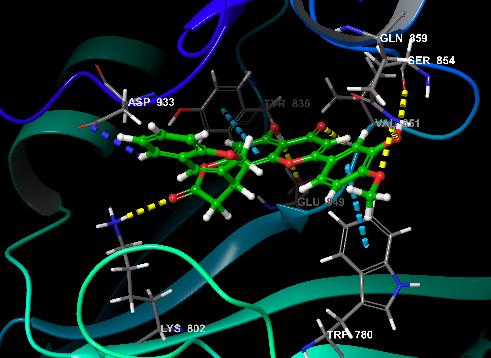 |
|  | STOCK1N-85998 | -1926.33 | 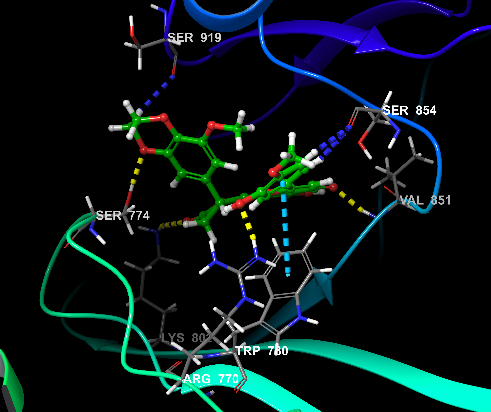 |
|  | STOCK1N-86351 | -1927.94 | 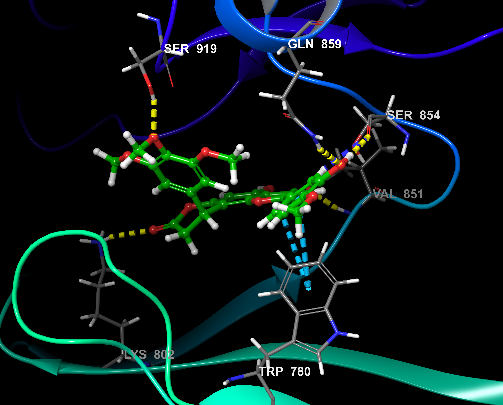 |
|  | STOCK1N-88743 | -1915.68 | 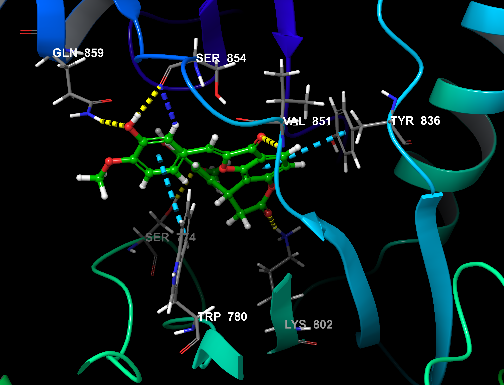 |
|  | STOCK1N-42002 | -1915.41 | 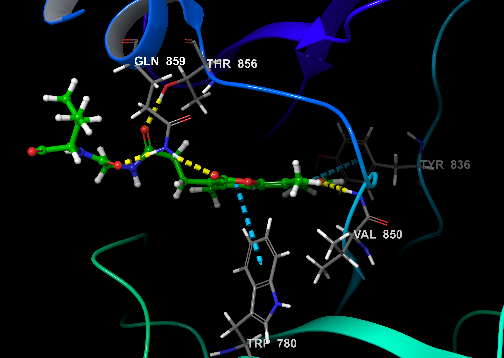 |
|  | STOCK1N-86060 | -1929.06 | 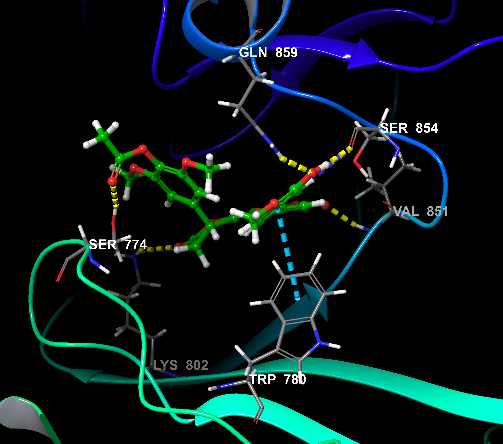 |
|  | STOCK1N-83010 | -1922.78 | 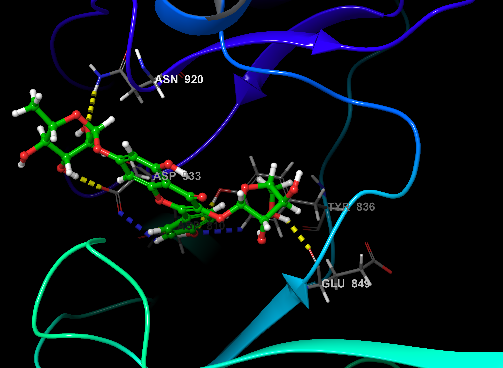 |


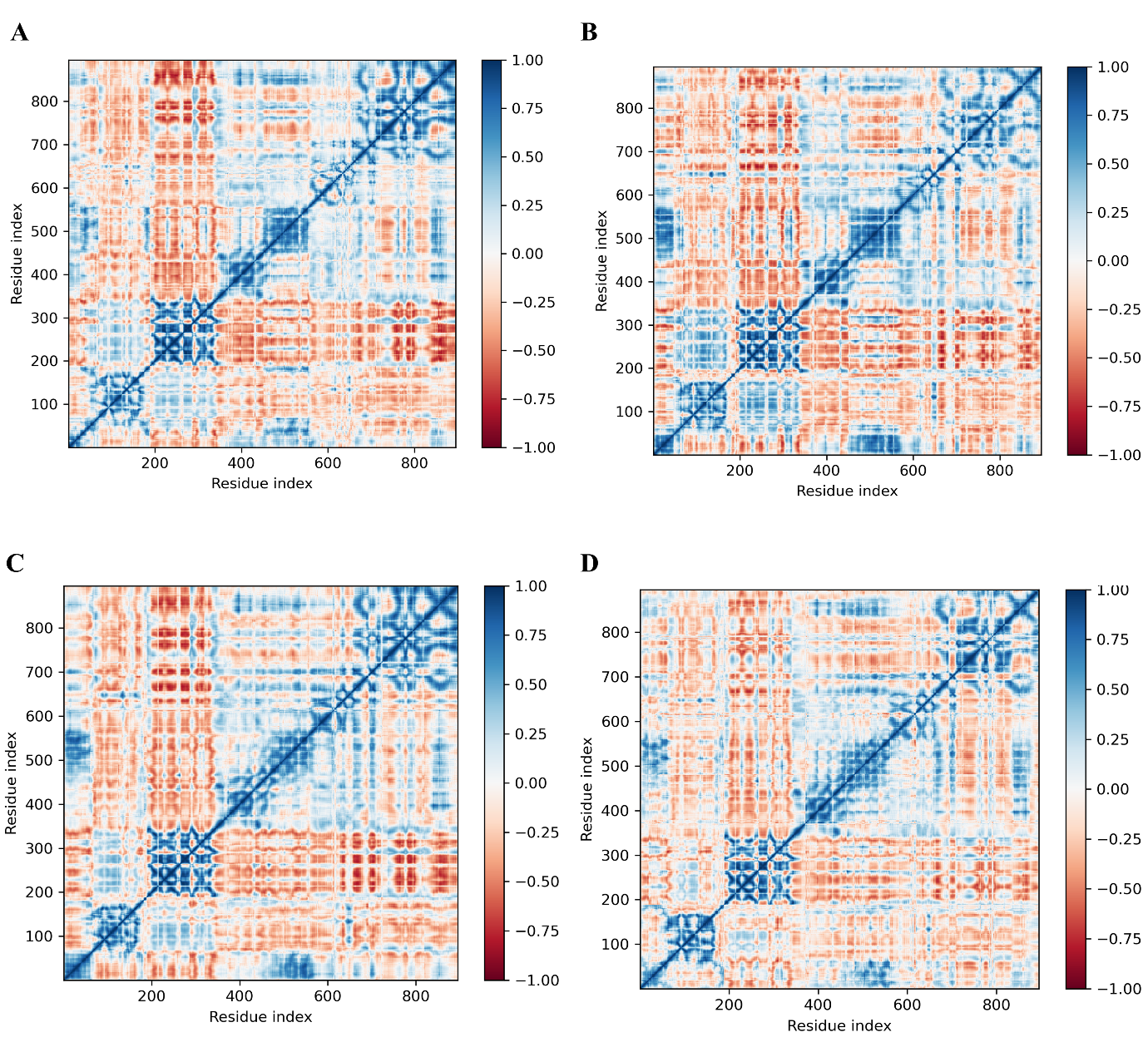


**Figure S1:** Cross-correlation of the Complexes.
